# LKB1IP promotes pathological cardiac hypertrophy by targeting PTEN/Akt signalling pathway

**DOI:** 10.1111/jcmm.16199

**Published:** 2021-01-24

**Authors:** Mi Tian, Xiuxin Jiang, Xinyun Li, Jianmin Yang, Cheng Zhang, Wencheng Zhang

**Affiliations:** ^1^ The Key Laboratory of Cardiovascular Remodeling and Function Research Chinese Ministry of Education The State and Shandong Province Joint Key Laboratory of Translational Cardiovascular Medicine Department of Cardiology Chinese National Health Commission and Chinese Academy of Medical Sciences Qilu Hospital Cheeloo College of Medicine Shandong University Jinan China; ^2^ Department of General Surgery Qilu Hospital of Shandong University Jinan China

**Keywords:** Akt, LKB1IP, pathological cardiac hypertrophy, PTEN

## Abstract

Pathological cardiac hypertrophy represents a leading cause of morbidity and mortality worldwide. Liver kinase B1 interacting protein 1 (LKB1IP) was identified as the binding protein of tumour suppressor LKB1. However, the role of LKB1IP in the development of pathological cardiac hypertrophy has not been explored. The aim of this study was to investigate the function of LKB1IP in cardiac hypertrophy in response to hypertrophic stimuli. We investigated the cardiac level of LKB1IP in samples from patients with heart failure and mice with cardiac hypertrophy induced by isoproterenol (ISO) or transverse aortic constriction (TAC). LKB1IP knockout mice were generated and challenged with ISO injection or TAC surgery. Cardiac function, hypertrophy and fibrosis were then examined. LKB1IP expression was significantly up‐regulated on hypertrophic stimuli in both human and mouse cardiac samples. LKB1IP knockout markedly protected mouse hearts against ISO‐ or TAC‐induced cardiac hypertrophy and fibrosis. LKB1IP overexpression aggravated ISO‐induced cardiomyocyte hypertrophy, and its inhibition attenuated hypertrophy in vitro. Mechanistically, LKB1IP activated Akt signalling by directly targeting PTEN and then inhibiting its phosphatase activity. In conclusion, LKB1IP may be a potential target for pathological cardiac hypertrophy.

## 1 INTRODUCTION

Heart failure is a leading cause of morbidity and mortality in modern society. According to the Atherosclerosis Risk in Communities Study of the US National Heart, Lung, and Blood Institute, the 30‐day, 1‐year and 5‐year case fatality rates after hospitalization for heart failure were 10.4%, 22% and 42.3%.^1^ Pathological cardiac hypertrophy is a key risk factor for heart failure.^2^ Multiple signalling pathways have been demonstrated to positively regulate protein synthesis and cardiac hypertrophy, including phosphoinositide 3‑kinase (PI3K)/Akt signalling, mitogen‐activated protein kinase signalling, Ca^2+^/calmodulin‐dependent kinase II signalling and calcineurin‐nuclear factor of activated T cell signalling.^3^, ^4^

Akt phosphorylates various intracellular substrates, thereby affecting metabolism, protein synthesis and cell survival/apoptosis. For example, Akt and subsequent phosphorylation of GSK3β predominantly mediate cardiac hypertrophy.^5^ Therefore, Akt protein has a direct impact on the development of cardiac hypertrophy. Phosphatase and tensin homology deleted on chromosome 10 (PTEN) is a tumour suppressor gene that dephosphorylates phosphatidylinositol (3,4,5)‐trisphosphate (PIP3) and depresses the PI3K‐Akt pathway.^6^ Loss of PTEN in the myocardium induces cardiac hypertrophy, which suggests the important role of PTEN in the development of cardiac hypertrophy.^7^ However, much less is known about the upstream regulatory mechanism of PTEN expression and activity.

Liver kinase B1 (LKB1) interacting protein 1 (LKB1IP; also known as LIP1 or STK11IP) consists of 25 exons; it maps to human chromosome 2q36 and encodes a leucine‐rich repeat‐containing protein of 121 kDa. LKB1IP appears to be a cytoplasmically located protein that may regulate LKB1 function by controlling its subcellular localization.^8^ LKB1IP also binds to the common mediator Smad4 protein of the transforming growth factor β and bone morphogenetic protein pathways.^9^ However, the role of LKB1IP in vivo, especially in regulating cardiac hypertrophy, remains unknown.

In this study, we used LKB1IP‐knockout mice and found that LKB1IP, by targeting PTEN, acts as a novel positive regulator of pathological cardiac hypertrophy induced by isoproterenol (ISO) or pressure overload. We identified a new role for LKB1IP in the development of cardiac hypertrophy.

## 2 MATERIALS AND METHODS

### 2.1 Animals

LKB1IP‐knockout mice (LKB1IP^‐/‐^) in a C57BL/6J background were purchased from the Jackson Laboratory (Cat No. 028 999). DNA was isolated from mouse tails and underwent PCR analysis with the primers 5’‐CAGTGTGCTACAGCCAGAGAG‐3’, 5’‐GAGCTGGGGAGGAGGTAGAC‐3’ and 5’‐AGGCCATCTCTCTGTCCTCA‐3’. Wild‐type (WT) C57BL/6J mice were controls. All mice were housed under specific pathogen‐free conditions on a 12‐h light/12‐h dark cycle with food and water freely available. The animal experiment was approved by the Animal Care Committee of Shandong University and was performed in compliance with the Animal Management Rules of the Chinese Ministry of Health (Document No. 55, 2001). All procedures conformed to the guidelines from the NIH Guide for the Care and Use of Laboratory Animals.

### 2.2 Human heart samples

Samples of failing hearts were from patients with end‐stage heart failure undergoing cardiac transplantation. Control tissues were taken from healthy heart organ donors, when the organ was not eligible for transplantation. The use of hearts samples from patients was approved by the Medical Institutional Ethics Committee of Qilu Hospital, Shandong University, China. The participants gave written informed consent prior to the inclusion in the study. The investigation conformed to the principles outlined in the 1964 Declaration of Helsinki and its later amendments.

### 2.3 Reagents

Isoproterenol (ISO), angiotensin II (AngII) and phenylephrine (PE) were from Sigma (St Louis, MO). The antibodies used were specific for LKB1IP (S7973‐58W, US Biological), PTEN (9188, Cell Signaling Technology), total Akt (4685, Cell Signaling Technology), phospho‐Akt (Thr308) (13 038, Cell Signaling Technology), HA‐tag (AHP1075, Bio‐Rad), Flag (SAB4301135, Sigma‐Aldrich), α‐SMA(,ab14106, Abcam), FITC conjugated isolectin B4 (BSI‐B4, L2895, Sigma‐Aldrich) and GAPDH (2118, Cell Signaling Technology). Adenoviral constructs to overexpress Flag‐tagged LKB1IP, HA‐tagged PTEN and GFP control were from ViGene BioSciences (Jinan, China). Control small interfering RNA (SC‐36869) and LKB1IP siRNA (SC‐146738) were from Santa Cruz Biotechnology.

### 2.4 Cell culture

Primary neonatal rat cardiomyocytes (NRCMs)NRCMs were prepared from the hearts of 2‐ to 3‐day‐old SD rats according to the following protocol.^10^ Briefly, PBS containing 0.03% trypsin and 0.04% collagenase type II was used to isolate cardiomyocytes, followed by fibroblast removal using a differential attachment technique. After removing fibroblasts, NRCMs were seeded at a density of 2 × 10^5^ cells per well onto six‐well culture plates coated with gelatin in plating medium consisting of DMEM/F12 medium supplemented with 20% foetal calf serum, BrdU (0.1 mmol/L, to inhibit the proliferation of fibroblasts) and penicillin/streptomycin at 37°C in 5% CO_2_. Transient transfection of siRNA targeting LKB1IP (siLKB1IP) in cardiomyocytes involved using Lipofectamine RNAiMAX (Invitrogen) to knock down LKB1IP according to the manufacturer's protocol. LKB1IP siRNA is a pool of 3 different siRNA duplexes: sc‐146738A, sc‐146738B and 146738C. The sequences were sense: CAUCUCGAUGUGGCCUAUAtt and antisense: UAUAGGCCACAUCGAGAUGtt for sc‐146738A; sense: CUAGAGUCCGAGACUGAAAtt and antisense: UUUCAGUCUCGGACUCUAGtt for sc‐146738B; sense: GUGUCAUGGGUAGUAUGUAtt and antisense: UACAUACUACCCAUGACACtt for sc‐146738C. LKB1IP adenoviruses were used to overexpress LKB1IP protein level.

### 2.5 Establishment of cardiac hypertrophy

We used male mice 8 to 10 weeks old at 2 weeks after transverse aortic constriction (TAC) or sham surgery or those subcutaneously injected with β‐adrenoceptor (β‐AR) agonist ISO (10 mg/kg per day, 14 days) or saline for 14 days, as described previously.^11^ Echocardiography and haemodynamic analyses were performed before mice were killed; then, the hearts were harvested to analyse the hypertrophic response.

### 2.6 Echocardiography

Echocardiography measurements were performed with a high‐resolution microimaging system equipped with a 30‐MHz transducer (Vevo2100; VisualSonics Inc) at the indicated times to evaluate the cardiac function of mice. Mice were anaesthetized with 1.5% to 2% inhaled isoflurane inhalation, and heart rates were kept at 400‐430 beats/min. Then, cardiac echocardiography was recorded on a heating plate at 37°C. M‐mode cardiac images of the left ventricle (LV) from the left parasternal long axis view at the papillary muscle level were used to assess the LV wall thickness and LV dimensions including interventricular septal thickness at end diastole (IVSd), interventricular septal thickness at end systole (IVSs), LV posterior wall thickness at end diastole (LVPWd), LV posterior wall thickness at end systole (LVPWs), end‐diastolic dimensions of the LV (LVEDd) and end‐systolic dimensions of the LV (LVEDs). Fractional shortening and LV transversal area in diastole and systole were calculated by using Vevo 770 V2.2.3.

### 2.7 Histopathology

Heart tissues were arrested with a 10% potassium chloride solution at end diastole, then fixed in 4% paraformaldehyde and maintained at 4°C until use. The fixed tissues were dehydrated and processed for paraffin embedding, and 5‐μm sections were stained with haematoxylin and eosin (H&E) or tetramethyl rhodamine isothiocyanate‐conjugated wheat germ agglutinin (WGA) (L4895, Sigma) and nuclei with 4',6‐diamidino‐2‐phenylindole (DAPI) (Ab104139, Abcam) to measure myocyte cross‐sectional areas. The degree of collagen deposition was detected by using a picrosirius red staining kit (ab150681, Abcam). The LV collagen volume was measured as the area of the picrosirius red positive‐stained cardiac field and was expressed as a percentage of the total area. Immunohistochemical staining was performed with LKB1IP antibody (C157105, LS bio, 1:200). Images were analysed by using Image‐Pro Plus 6.0.

### 2.8 Immunofluorescence

For immunocytochemical analysis, NRCMs were infected with adenoviruses for 24 hr or siRNA for 48 hr and then stimulated with ISO for 24 hr Cells were fixed with 4% formaldehyde in phosphate‐buffered saline (PBS) for 15 min at room temperature, permeabilized with 0.1% Triton X‐100 in PBS for 10 min, incubated with FITC‐labelled Phalloidin (40735ES75; Yeasen) and mounted with DAPI (Ab104139; Abcam). Images of the stained cells were acquired by fluorescence microscopy (BX41, Olympus, Japan) and confocal microscopy (ZEISS) and analysed by using Image‐Pro Plus 6.0. We observed 30 cells in randomly selected fields for each group to quantitatively analyse cell sizes.

### 2.9 Immunoprecipitation

Cells were lysed in an ice‐cold immunoprecipitation buffer (NP‐40 lysis buffer) containing protease inhibitor cocktail tablets (539 131, Millipore) and centrifuged at 13,000 × g for 15 min. The obtained cell lysates were incubated with the indicated antibody on a rocking platform at 4 ℃ overnight. On the second day, cell lysates were incubated with protein A/G‐agarose beads (SC2003; Santa Cruz Biotechnology) for 2 hr at 4℃. The immune complex was collected after washing with cold immunoprecipitation buffer and subjected to immunoblotting with the indicated primary antibodies and corresponding secondary antibodies.

### 2.10 Western blot analysis

Protein extracts were prepared by lysing tissues or cells in RIPA lysis buffer, fractionated by SDS‐PAGE and then transferred onto PVDF membranes. After blocking in 5% skim milk, blots were probed with specific primary antibodies. The membranes were immunoblotted with a primary antibody at 4℃ overnight. After 3 cycles of cleaning with TBST, membranes were incubated with appropriate horseradish peroxidase‐conjugated secondary antibodies and observed by enhanced chemiluminescence (Pierce).

### 2.11 Quantitative PCR (qPCR)

Total RNA was extracted from hearts or cells by using TRIzol reagent (Invitrogen, Carlsbad, CA). An amount of 1 μg RNA was reverse‐transcribed into cDNA by using the PrimeScript RT Reagent Kit (Takara Biomedical Technology). PCR amplification involved using the SYBR PCR mix (Takara Biomedical Technology Co.). The primers for quantitative PCR were for mouse‐LKB1IP, 5’‐TTAGACAGCTCCCTGCGTCT‐3’ and 5’‐TCAGGAAGCCTTTGCAGTCC‐3’; mouse‐ANP, 5’‐CTCCGATAGATCTGCCCTCTTGAA‐3’ and 5’‐GGTACCGGAAGCTGTTGCAGCCTA‐3’; mouse‐BNP, 5’‐GCTCTTGAAGGACCAAGGCCTCAC‐3’ and 5’‐GATCCGATCCGGTCTATCTTGTGC‐3’; mouse‐β‐MHC, 5’‐AACCTGTCCAAGTTCCGCAAGGTG‐3’ and 5’‐GAGCTGGGTAGCACAAGAGCTACT‐3’, mouse‐GAPDH, 5'‐TTGTCAAGCTCATTTCCTGGTATG‐3' and 5'‐GCCATGTAGGCCATGAGGTC‐3'; rat‐LKB1IP, 5’‐CTACGATGAGGTGTCTCGGC‐3’ and 5’‐AGAGCCTCCTGGGTTACTGT‐3’; rat‐ANP, 5’‐CATGGGCTCCTTCTCCATCA‐3’ and 5’‐TGGCCTGGGAGCCAAA‐3’; rat‐BNP, 5’‐CGGGCTGAGGTTGTTTTAGG‐3’ and 5’‐GCCGCAGGCAGAGTCAGA‐3’; rat‐β‐MHC, 5’‐TTGGCACGGACTGCGTCATC‐3’ and 5’‐GAGCCTCCAGAGTTTGCTGAAGGA‐3’; rat‐GAPDH, 5’‐GACATGCCGCCTGGAGAAAC‐3’ and 5’‐AGCCCAGGATGCCCTTTAGT‐3’.

### 2.12 Statistical analysis

All data are expressed as mean ± SEM. Analyses involved use of SPSS v23 (SPSS Inc, Chicago, IL) and data passed normality and equal variance tests. PASS 11 (NCSS Inc) was used to calculate the sample size. Statistical comparisons between 2 groups involved Student's t test and otherwise one‐way ANOVA and Bonferroni post‐tests. All statistical tests were two‐tailed, and *P* < .05 was considered statistically significant.

## 3 RESULTS

### 3.1 LKB1IP expression is up‐regulated in the hypertrophic heart and cells

**FIGURE 1:**
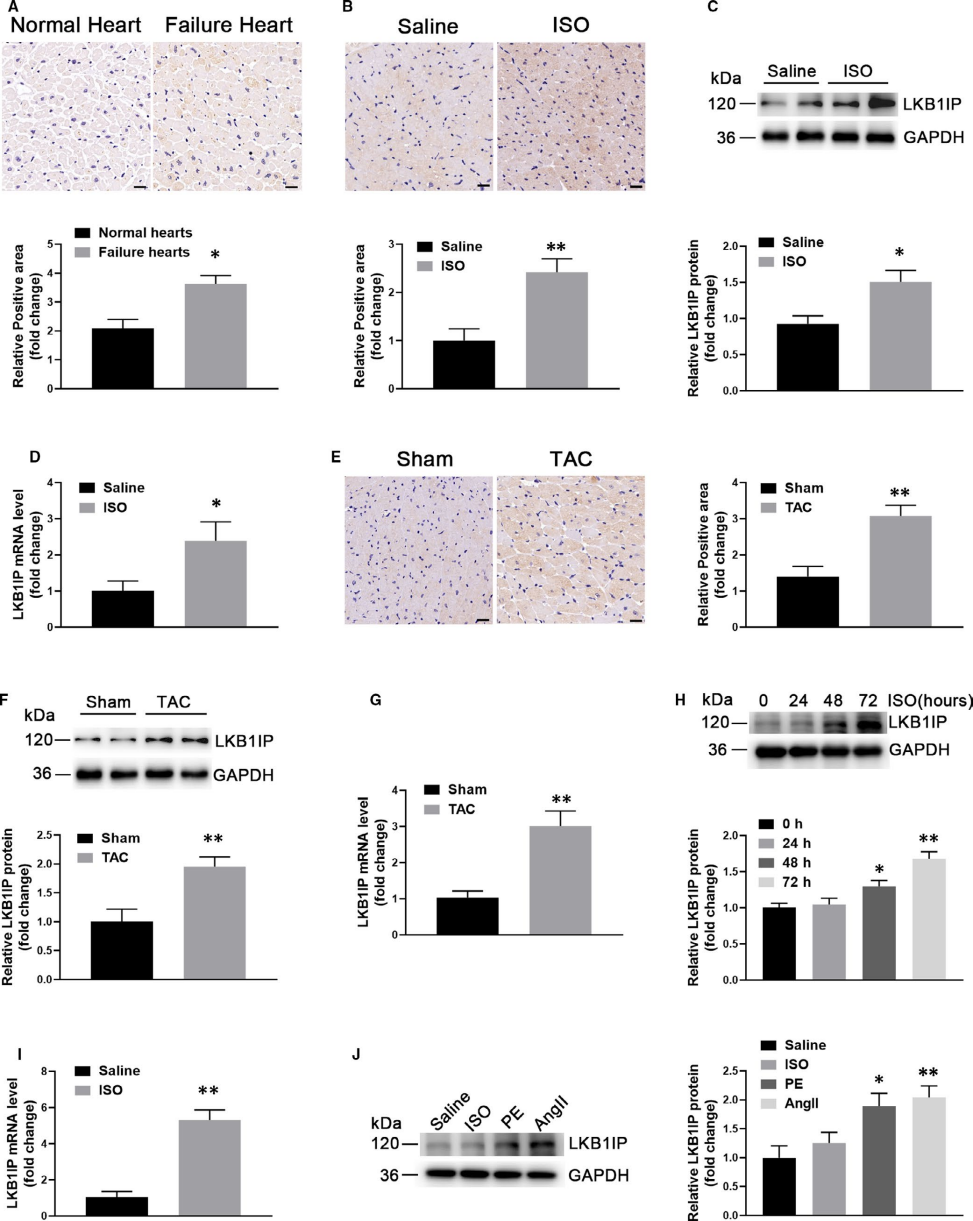
LKB1IP expression is up‐regulated in hypertrophic hearts and cells. (A) Immunohistochemical staining of LKB1IP protein in heart tissue from normal human controls and heart failure patients (n = 5). *P < .05 *vs* normal control. Scale bar, 20 μm. **(B)** Immunohistochemical staining of LKB1IP protein in heart tissue from control and ISO‐induced hypertrophic mice (n = 5). **P < .01*vs* saline. Scale bar, 20 μm. **(C)** Western blot analysis of LKB1IP protein expression in control and ISO‐induced hypertrophic mouse hearts (n = 5). *P < .05 *vs* saline. **(D)** Quantitative PCR analysis of LKB1IP mRNA level from hearts of control and ISO‐induced cardiac hypertrophic mice (n = 5). *P < .05 *vs* saline. **(E)** Immunohistochemical staining of LKB1IP protein in mouse heart samples at 2 weeks after transverse aortic constriction (TAC) or sham surgery (n = 5). **P < .001 *vs* sham. Scale bar, 20 μm. **(F)** Western blot analysis of LKB1IP protein expression in control or TAC‐induced hypertrophic mouse hearts at 2 weeks (n = 4). **P < .01*vs* sham. **(G)** Quantitative PCR analysis of LKB1IP mRNA levels in hearts of sham and TAC‐induced cardiac hypertrophic mice (n = 4). **P < .01 *vs* sham. **(H)** Western blot analysis of LKB1IP protein expression in NRCMs treated with 10 μmol/L ISO at the indicated times (n = 3). *P < .05, **P < .01 *vs* 0 hr **(I)** Quantitative PCR analysis of LKB1IP mRNA level in the NRCMs treated with 10 μmol/L ISO for 48 hr (n = 3). **P < .01*vs* saline. **(J)** Western blot analysis of LKB1IP protein expression in NRCMs treated with 10 μmol/L ISO, 100 nmol/L AngII or 100 μmol/L phenylephrine (PE) for 24 hr (n = 3). *P < .05 **P < .01 *vs* saline

To explore the role of LKB1IP in the development of cardiac hypertrophy and heart failure, we first investigated LKB1IP abundance in human and mouse hypertrophic heart disease. Immunohistochemistry revealed significantly greater expression of LKB1IP in the human failing heart than in normal controls (*P* < .05; Figure 1A). The baseline clinical characteristics of healthy donors and heart failure patients were shown in Supplementary Table 1. Mouse cardiac hypertrophy was induced by infusing the hypertrophic agonist ISO or by TAC surgery. LKB1IP was up**‐**regulated in immunohistochemical‐stained hypertrophic mouse heart sections as compared with corresponding controls (Figure 1B and 1E), and the protein and mRNA levels of LKB1IP were also significantly elevated (Figure 1C, 1D, 1F and 1G). Similarly, LKB1IP protein and mRNA levels were increased in hypertrophic cardiac cells, that is, NRCMs stimulated with ISO (Figure 1H and 1I). Furthermore, the expression of LKB1IP was up**‐**regulated under pathological hypertrophic stimuli such as AngII or PE for 24 hr (Figure 1J). Collectively, these data demonstrate elevated LKB1IP expression under pathological cardiac hypertrophy.

### 3.2 Establishment and analysis of LKB1IP knockout mice

**FIGURE 2:**
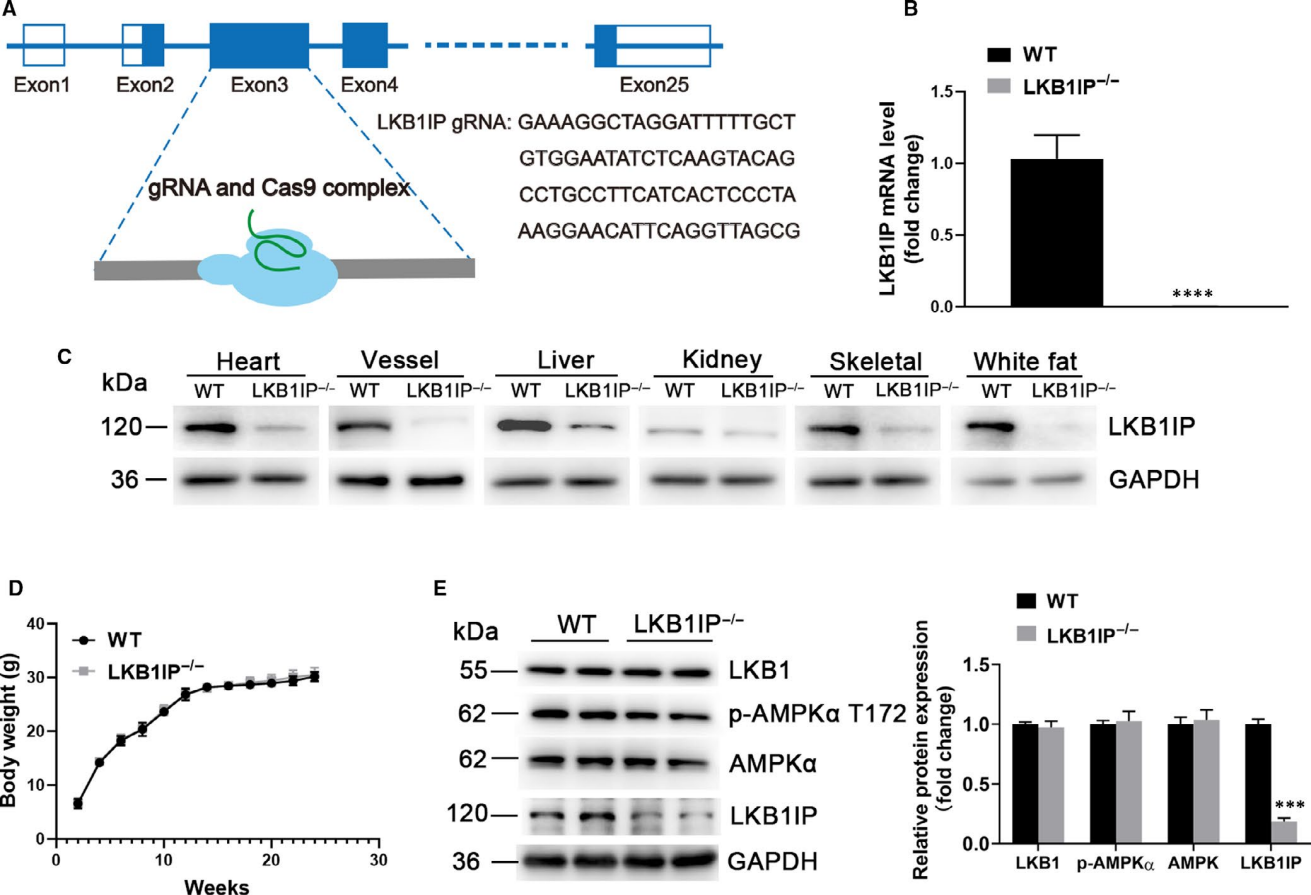
**Establishment and analysis of LKB1IP knockout mice model. (A)** Schematic diagram of CRISPR/Cas9 to knock out LKB1IP. **(B)** Quantitative PCR analysis of LKB1IP mRNA level in the hearts of wild‐type (WT) (n = 3) and LKB1IP^‐/‐^ mice (n = 5). ****P < .0001 *vs* WT. **(C)** Western blot analysis of LKB1IP protein expression in tissues of WT and LKB1IP^‐/‐^ mice. **(D)** Bodyweight of WT and LKB1IP^‐/‐^ mice (n = 5). **(E)** Western blot analysis of LKB1, p‐AMPKα T172 and AMPKα protein expression in hearts of WT and LKB1IP^‐/‐^ mice(n = 3). ***P < .001 *vs* WT

To evaluate the hypertrophic effects of LKB1IP in vivo, we first used a mouse model with global knockout of LKB1IP (LKB1IP^‐/‐^). Four guide RNAs (GAAAGGCTAGGATTTTTGCT, GTGGAATATCTCAAGTACAG, CCTGCCTTCATCACTCCCTA and AAGGAACATTCAGGTTAGCG) were designed to delete 479 bp in exon 3 of LKB1IP gene, and Cas9 nuclease was introduced into C57BL/6NJ‐derived fertilized eggs with well‐recognized pronuclei followed by transfer to pseudopregnant females (Figure 2A). The absence of LKB1IP in the heart tissues was first confirmed by real‐time PCR (Figure 2B). LKB1IP expression in the main heart tissues was further detected by Western blot analysis, thus indicating effective LKB1IP deletion in LKB1IP^‐/‐^ mice (Figure 2C). Wild‐type (WT) and LKB1IP^‐/‐^ mice did not differ in bodyweight (Figure 2D). The blood pressure of WT and LKB1IP^‐/‐^ mice was measured. There were no significant differences between WT and LKB1IP^‐/‐^ mice (Supplementary Figure 4). To clarify the effect of LKB1IP knockout on LKB1 and its downstream AMP‐activated protein kinase (AMPK), we assessed protein levels by Western blot analysis, which showed no significant differences in LKB1, phosphorylated AMPKα (T172) and total AMPKα levels between WT and LKB1IP ^‐/‐^ mice (Figure 2E).

### 3.3 LKB1IP deficiency alleviates ISO‐induced hypertrophy in vivo

**FIGURE 3:**
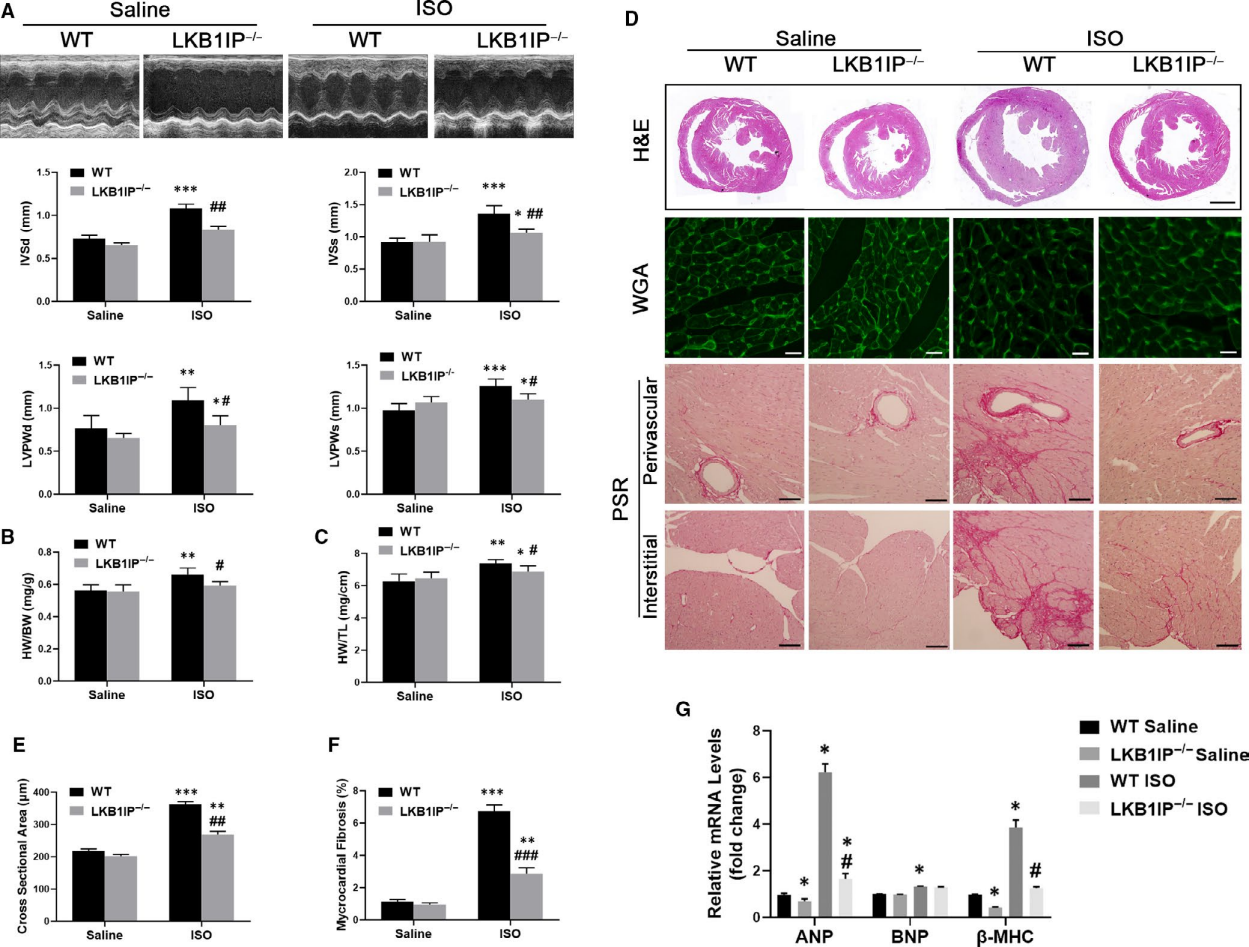
**LKB1IP deficiency alleviates ISO‐induced cardiac hypertrophy. (A)** Representative M‐mode echocardiography of the left ventricle (top). Measurement of diastolic interventricular septal thickness (IVSd), systolic interventricular septal thickness (IVSs), diastolic left ventricular posterior wall (LVPWd) and systolic left ventricular posterior wall (LVPWs) in the indicated groups (n = 5). *P < .05, **P < .01 ***P < .001 *vs* WT Saline; ^#^ *P* < .05, ^##^ *P* < .01 *vs* WT ISO. **(B)** Quantitative analysis of ratio of heart weight to bodyweight (HW/BW) in the indicated groups (n = 5). **P < .01 *vs* WT Saline; ^#^ *P* < .05 *vs* WT ISO. **(C)** Quantitative analysis of ratio of HW to tibial length (TL) in the indicated groups (n = 5). *P < .05, **P < .05 *vs* WT Saline; ^#^ *P* < .05 *vs* WT ISO. **(D)** Analysis of whole hearts (the first row; scale bar, 1,000 μm) and heart sections stained with wheat germ agglutinin (WGA; the second row; scale bar, 50 μm) or picosirius red (PSR; the third and fourth rows; scale bars, 50 μm) from the indicated groups 7 days after saline or ISO treatment. **(E)** Quantitative analysis of the average cardiomyocyte cross‐sectional area in the indicated groups, n > 100 cells per group. **P < .01, *** *P* < .001 *vs* WT Saline; ^##^ *P* < .01*vs* WT ISO. **(F)** Quantitative analysis of left ventricle (LV) collagen volume in the indicated groups, n > 15 fields per group. **P < .01, *** *P* < .001 *vs* WT Saline; ^###^ *P* < .001 *vs* WT ISO. **(G)** Quantitative PCR analysis of ANP, BNP and β‐MHC mRNA levels in the indicated groups (n = 3). *P < .05, *** *P* < .001 *vs* WT Saline; ^#^ *P* < .05 *vs* WT ISO

We injected the β‐adrenoceptor (β‐AR) agonist ISO (10 mg/kg per day, 14 days) in mice to establish the hypertrophy model. LKB1IP^‐/‐^ mice did not show any apparent differences in heart morphology or function at baseline as compared with WT mice. On day 14, echocardiography and haemodynamic analyses were performed for the final time before mice were killed. As expected, on day 14, WT mice showed a hypertrophic phenotype, as indicated by a significant increase in echocardiography measurements including diastolic interventricular septal thickness (IVSd), systolic interventricular septal thickness (IVSs), diastolic left ventricular posterior wall (LVPWd) and systolic left ventricular posterior wall (LVPWs) (Figure 3A). The results showed no significant differences in ejection fraction (EF) and fraction shortening (FS) (Supplementary Table 2). Furthermore, ratios of heart weight (HW) to bodyweight (HW/BW; Figure 3B) and HW to tibia length (HW/TL; Figure 3C) were significantly increased. However, ISO‐induced cardiac hypertrophy was significantly attenuated in LKB1IP^‐/‐^ mice, as demonstrated by lower IVSd, IVSs, LVPWd and LVPWs and ratios of HW/BW and HW/TL in LKB1IP^‐/‐^ than in WT mice (Figure 3A‐3C). The myocardial tissue of WT mice subjected to ISO treatment showed a hypertrophic myocardial area upon H&E staining and elevated cardiomyocyte area upon wheat germ agglutinin (WGA) staining, which were attenuated in ISO‐treated LKB1IP^‐/‐^ mice (Figure 3D).

We next assessed ISO‐induced cardiac fibrosis, a classical feature in the development of pathological cardiac hypertrophy. Paraffin‐embedded slides were stained with picrosirius red to determine the extent of fibrosis. Both interstitial and perivascular fibrosis were markedly increased in ISO‐treated WT hearts but were greatly decreased in LKB1IP^‐/‐^ hearts (Figure 3E and 3F). The expression of cardiac hypertrophy‐related genes such as atrial natriuretic peptide (ANP), brain natriuretic peptide (BNP) and myosin heavy chain β (β‐MHC) was significantly increased in ISO‐treated WT mice (Figure 3G). ANP and β‐MHC levels in ISO‐induced hypertrophic cardiac tissues were lower in LKB1IP^‐/‐^ than WT mice (Figure 3G). However, BNP level did not significantly differ between WT and LKB1IP^‐/‐^ mice treated with ISO. Both capillary and arteriolar density were increased in mice treated with ISO compared with saline. However, there were no significantly differences between ISO‐treated WT and LKB1IP^‐/‐^ mice (Supplementary Figure 1A‐1B). These loss‐of‐function data indicate that LKB1IP deficiency alleviates the pathological cardiac remodelling induced by ISO.

### 3.4 LKB1IP deficiency alleviates pressure overload–induced cardiac hypertrophy in mice

**FIGURE 4:**
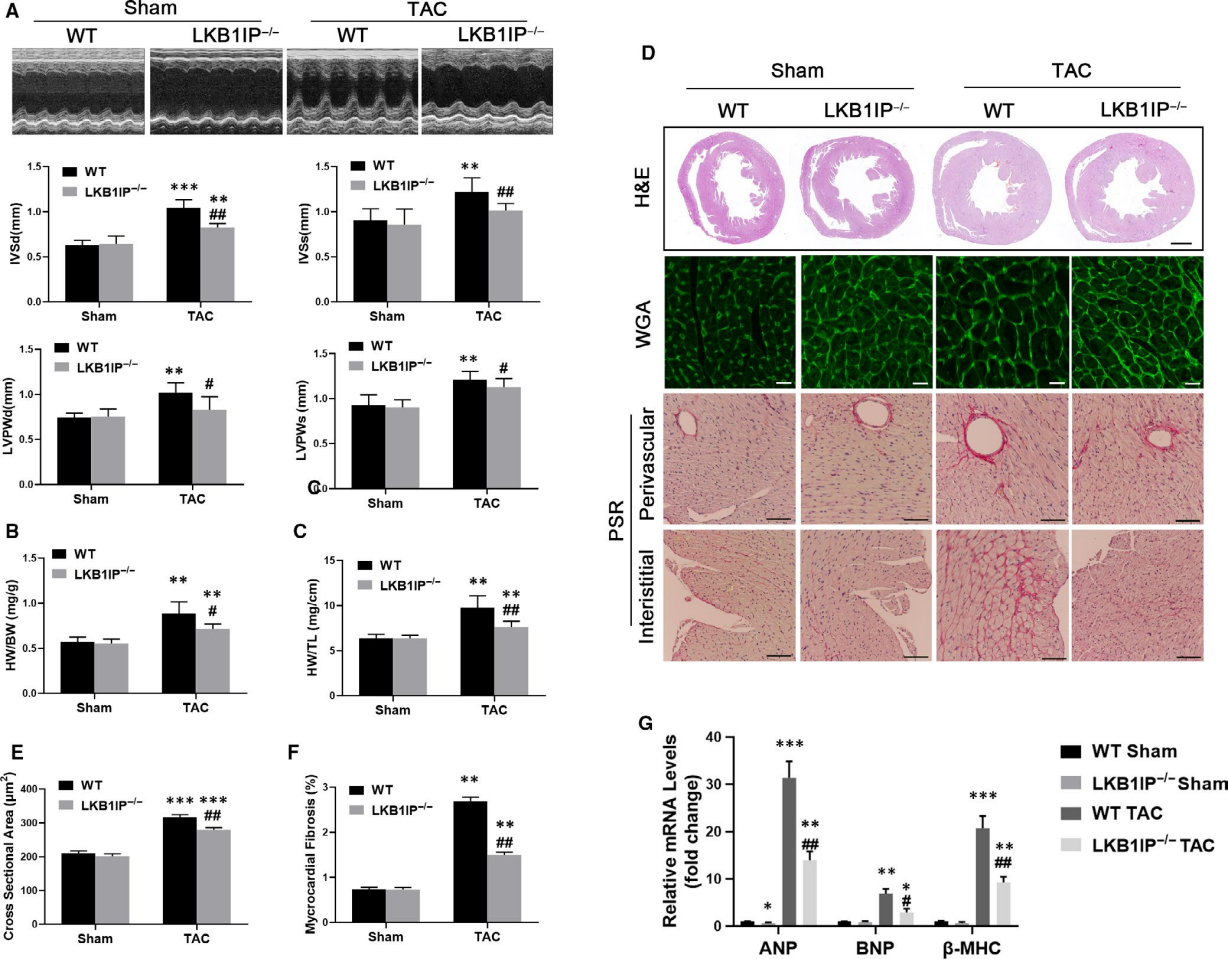
**LKB1IP deficiency alleviates TAC‐induced cardiac hypertrophy. (A)** Representative M‐mode echocardiography of the left ventricle (top). Measurement of diastolic interventricular septal thickness (IVSd), systolic interventricular septal thickness (IVSs), diastolic left ventricular posterior wall (LVPWd) and systolic left ventricular posterior wall (LVPWs) in the indicated groups (n = 5). **P < .01,***P < .001 *vs* WT Sham; ^#^ *P* < .05, ^##^ *P* < .01 *vs* WT TAC. Quantitative analysis of **(B)** ratio of HW/BW in the indicated groups (n = 5) and **(C)** ratio of HW/TL in the indicated groups (n = 5) **P < .01 *vs* WT Sham; ^#^ *P* < .05, ^##^ *P* < .01 *vs* WT TAC. **(D)** Analysis of whole hearts (the first row; scale bar, 1,000 μm) and heart sections stained with WGA (the second row; scale bar, 50 μm) or PSR (the third and fourth rows; scale bars, 50 μm) in the indicated groups at 14 days after sham or TAC surgery. **(E)** Quantitative analysis of the average cardiomyocyte cross‐sectional area in the indicated groups, n > 100 cells per group. ***P < .001 *vs* WT Sham; ^##^ *P* < .01 *vs* WT TAC. **(F)** Quantitative analysis of LV collagen volume in the indicated groups, n > 15 fields per group. **P < .01 *vs* WT Sham; ^##^ *P* < .01 *vs* WT TAC. **(G)** Quantitative PCR analysis of ANP, BNP and β‐MHC mRNA levels in the indicated groups (n = 3). *P < .05, **P < .01, ***P < .001 *vs* WT Sham; ^#^ *P* < .05, ^##^ *P* < .01 *vs* WT TAC

We further evaluated whether LKB1IP knockout could alleviate pressure overload‐induced cardiac hypertrophy in vivo. To this end, we applied TAC surgery to mice. At 2 weeks after TAC, WT mice showed cardiac hypertrophy, as demonstrated by increased left ventricular wall thickness and reduced HW/BW and HW/TL ratios (Figure 4A‐4C). Ejection fraction (EF) and fraction shortening (FS) were significantly decreased in mice with TAC (Supplementary Table 3). On H&E and WGA staining, TAC surgery increased cardiomyocyte size and perivascular and interstitial fibrosis in WT mice (Figure 4D‐4F). Remarkably, these hypertrophic features were alleviated in hearts of LKB1IP^‐/‐^ mice that underwent TAC (Figure 4D‐4F). Consistently, these hypertrophic pathological phenotypes were accompanied by up**‐**regulated hypertrophic genes including ANP, BNP and β‐MHC in WT mice with TAC, which was attenuated in LKB1IP^‐/‐^ mice (Figure 4G). Besides, both capillary and arteriolar density were significantly increased in TAC compared with sham groups. However, there were no differences between WT and LKB1IP^‐/‐^ mice underwent TAC (Supplementary Figure1C‐1D). Thus, LKB1IP knockout alleviated pressure overload‐induced cardiac hypertrophy in mice.

### 3.5 LKB1IP positively regulates ISO‐induced cardiomyocyte hypertrophy in vitro

**FIGURE 5:**
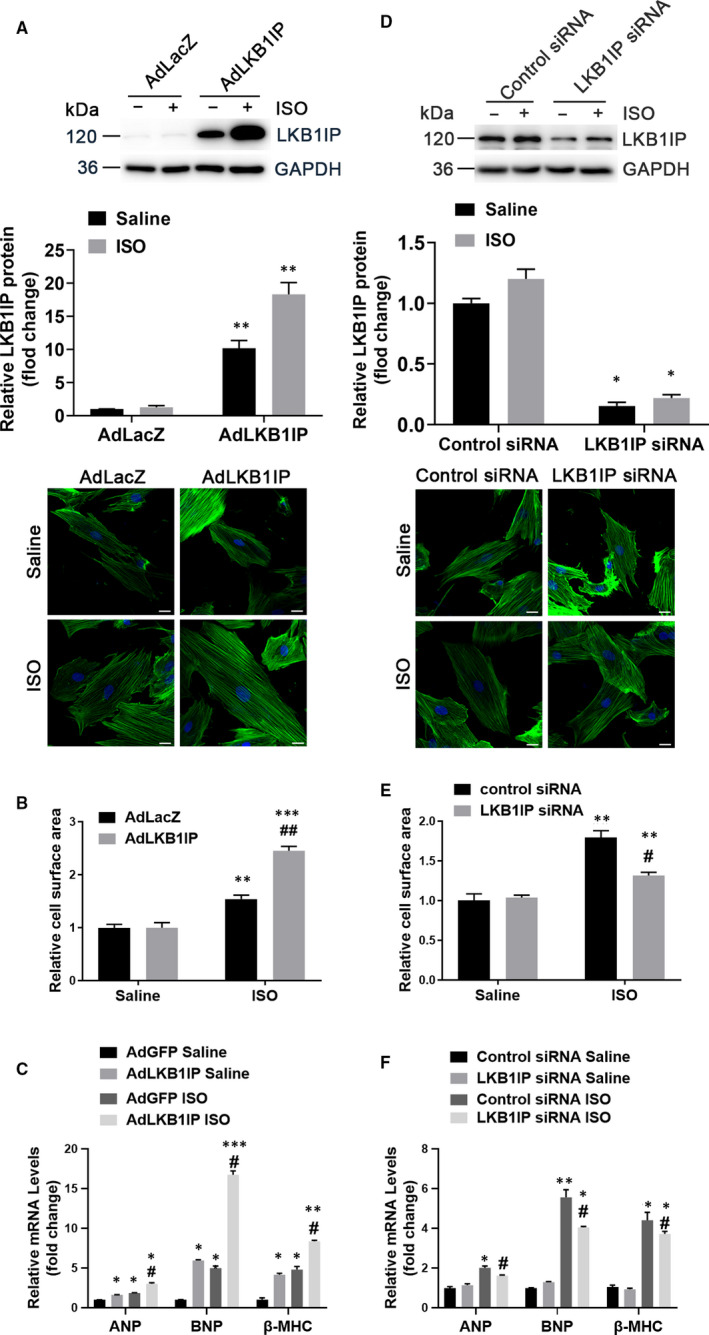
**LKB1IP positively regulates ISO‐induced cardiomyocyte hypertrophy in vitro. (A)** NRCMs were infected with adenovirus expressing LacZ or LKB1IP for 24 hr, then treated with saline or 10 μmol/L ISO for 24 hr, followed by α‐actinin staining(down). Scale bar, 20 μm. Western blot analysis of LKB1IP from NRCMs infected with adenovirus expressing LacZ or LKB1IP (up). **(B)** Quantitative analysis of NRCM size in each group (n > 50 cells per group). **P < .01, ***P < .001 *vs* AdLacZ Saline; ^##^ *P* < .01 *vs* AdLacZ ISO. **(C)** Quantitative PCR analysis of ANP, BNP and β‐MHC mRNA levels in NRCMs in each group (n = 3). *P < .05, **P < .01, ***P < .001 *vs* AdLacZ Saline; ^##^ *P* < .01 *vs* AdLacZ ISO. **(D)** NRCMs were transfected with control or LKB1IP siRNA for 24 hr, then treated with ISO for 24 hr, followed by α‐actinin staining(down). Scale bar, 20 μm. Western blot analysis of LKB1IP from NRCMs transfected with control or LKB1IP siRNA (up). **(E)** Quantitative analysis of NRCM size in each group (n > 50 cells per group). **P < .01 *vs* control siRNA Saline; ^#^ *P* < .05 *vs* control siRNA ISO. **(F)** Quantitative PCR analysis of ANP, BNP and β‐MHC mRNA levels in NRCMs in each group (n = 3). *P < .05, **P < .01, ***P < .01 *vs* control siRNA Saline; ^#^ *P* < .05, ^##^ *P* < .01 *vs* control siRNA ISO

We investigated whether LKB1IP directly regulates cardiomyocyte hypertrophy induced by ISO in NRCMs. The immunofluorescence against cTnI to identify the primary neonatal rat cardiomyocytes was shown in Supplementary Figure 3. NRCMs were infected with adenovirus expressing FLAG‐tagged LKB1IP for 48 hrs followed by immunohistochemistry against FLAG to detect the efficiency of transduction, which was about 70% (Supplementary Figure 2). As compared with PBS treatment, ISO treatment significantly increased the mean cross‐sectional area of NRCMs, which was further enhanced by adenoviral overexpression of LKB1IP (Figure 5A‐5B). In addition, ISO‐elevated ANP, BNP and β‐MHC mRNA expression were greatly enhanced with LKB1IP overexpression (Figure 5C). Consistently, ISO‐induced cardiomyocyte hypertrophy was reduced with LKB1IP siRNA knockdown (Figure 5D‐5E). Similarly, LKB1IP knockdown attenuated ISO‐elevated ANP, BNP and β‐MHC mRNA levels (Figure 5F). These data further demonstrate that LKB1IP positively regulated cardiomyocyte hypertrophy under hypertrophic stress.

### 3.6 LKB1IP positively regulates Akt phosphorylation

**FIGURE 6:**
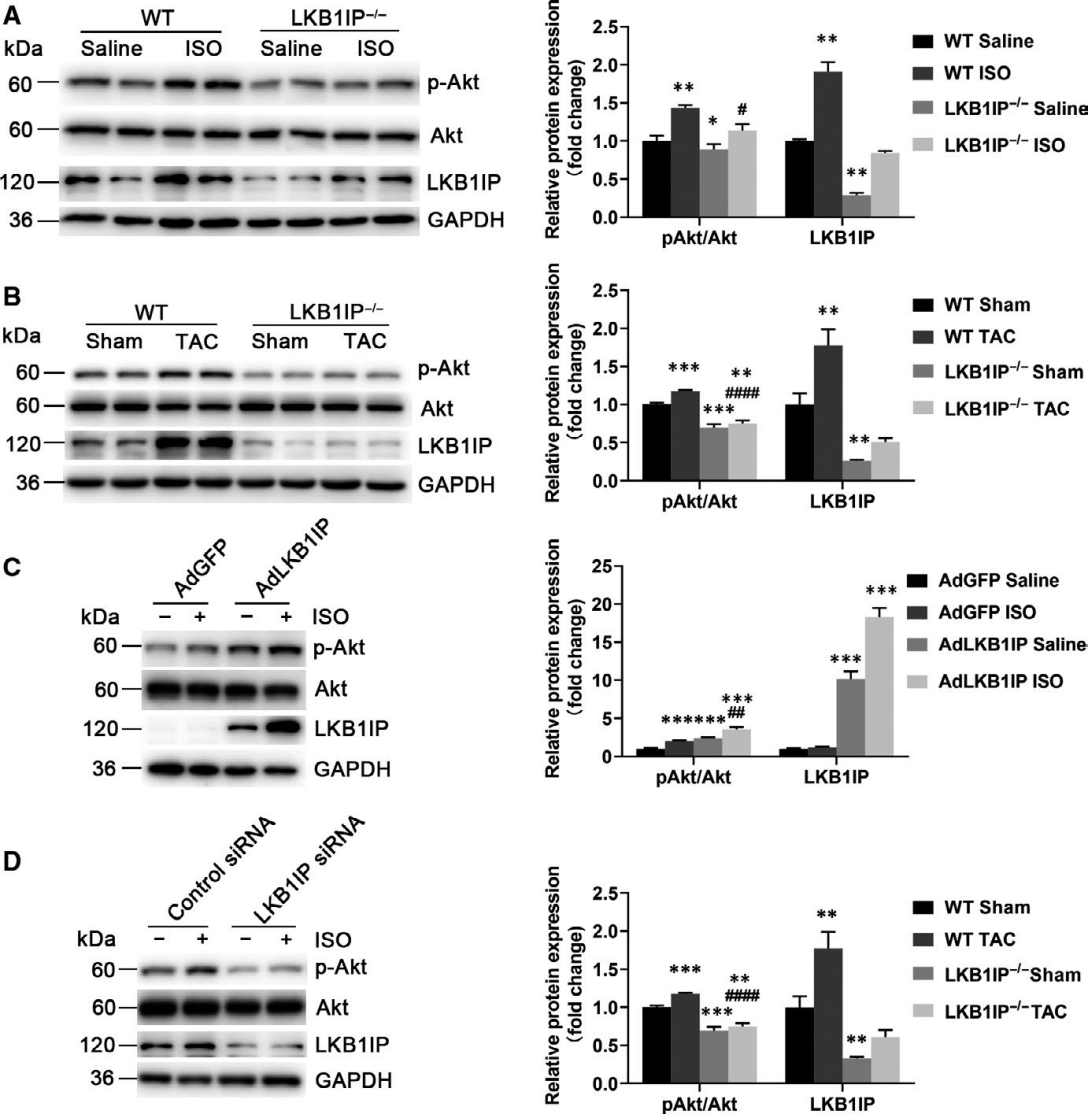
**LKB1IP positively regulates Akt phosphorylation. (A)** Western blot analysis of phosphorylated Akt from hearts of ISO‐infused WT and LKB1IP^‐/‐^ mice (n = 4). *P < .05, **P < .01*vs* WT Saline; ^#^ *P* < .05 *vs* WT ISO. (**B)** Western blot analysis of phosphorylated Akt level in hearts of WT and LKB1IP^‐/‐^ mice after TAC surgery (n = 4). **P < .01, ***P < .001*vs* WT Sham; ^####^ *P* < .0001 *vs* WT TAC. **(C)** Western blot analysis of phosphorylated Akt level in NRCMs infected with AdGFP or AdLKB1IP followed by ISO treatment (n = 3). ***P < .001 *vs* AdGFP Saline; ^##^ *P* < .01 *vs* AdGFP ISO. **(D)** Western blot analysis of phosphorylated Akt level in NRCMs transfected with control or LKB1IP siRNA followed by ISO treatment (n = 3). **P < .01, ****P < .0001 *vs* Control siRNA Saline; ^##^ *P* < .01 *vs* Control siRNA ISO

To explore the molecular mechanism by which LKB1IP regulates cardiac hypertrophy, we detected hypertrophy‐related signalling pathways. In the mouse models of cardiac hypertrophy, ISO or TAC significantly increased Akt phosphorylation at Thr 308 as compared with their controls in WT mice, which was attenuated in LKB1IP^‐/‐^ mice (Figure 6A‐6B). Consistently, ISO‐induced Akt phosphorylation in NRCMs was enhanced with LKB1IP overexpression (Figure 6C) or suppressed with LKB1IP knockdown (Figure 6D). We performed Western blot using NRCMs and mouse cardiac tissue lysis to detect the three isoforms of Akt. All the Akt isoforms have the similar molecular weight and could not be distinguished by Western blot with Akt antibody (Supplementary Figure 6). Thus, Akt signalling may be positively related to LKB1IP expression and LKB1IP may regulate ISO‐induced cardiac hypertrophy via the Akt signalling pathway.

### 3.7 LKB1IP promotes Akt phosphorylation via direct targeting PTEN

**FIGURE 7:**
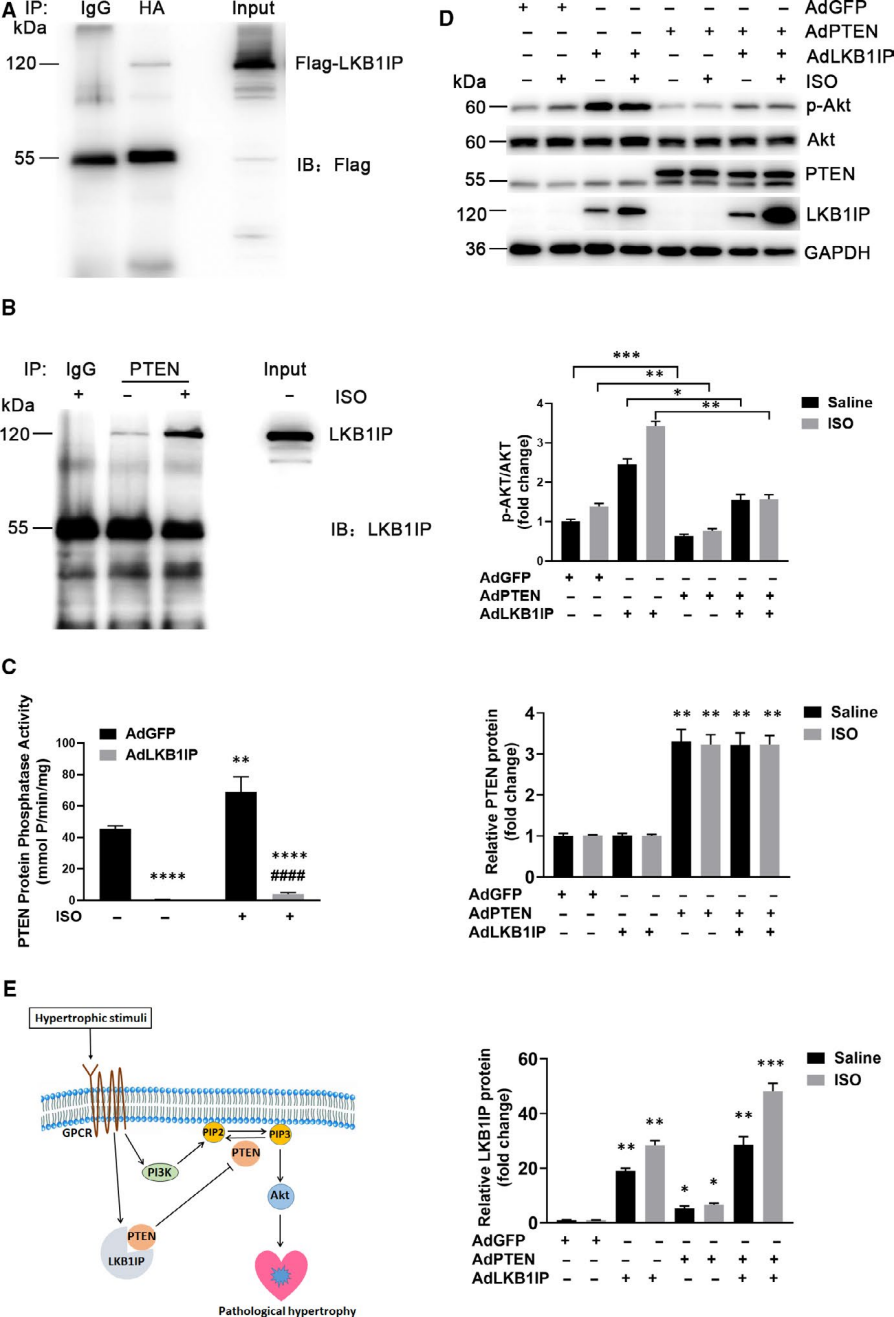
**LKB1IP promotes Akt phosphorylation by directly targeting PTEN. (A)** NRCMs were infected with adenovirus expressing Flag‐tagged LKB1IP and HA‐tagged PTEN followed by immunoprecipitation with anti‐HA antibody and Western blot analysis with anti‐Flag antibody. **(B)** NRCMs were treated with ISO for 15 min followed by immunoprecipitation with PTEN antibody and Western blot analysis with LKB1IP antibody. **(C)** NRCMs were infected with adenovirus expressing GFP or LKB1IP for 24 hr, then treated with saline or 10 μmol/L ISO for 15 min followed by PTEN phosphatase activity assay (n = 3). **P < .01, ****P < .0001 *vs* AdGFP Saline; ^####^ *P* < .0001 *vs* AdGFP ISO. **(D)** Western blot analysis of phosphorylated Akt level in NRCMs infected with adenovirus expressing GFP, LKB1IP or PTEN for 24 hr and treated ISO (n = 3). *P < .05, **P < .01, ***P < .001 *vs* AdGFP Saline. **(E)** Diagram for the function of LKB1IP in the development of pathological cardiac hypertrophy

To investigate how LKB1IP regulates Akt phosphorylation, we detected whether LKB1IP could interact with PTEN, the negative regulator of PI3K/Akt signalling. NRCMs were infected with adenovirus expressing Flag‐tagged LKB1IP and HA‐tagged PTEN followed by immunoprecipitation, which showed that LKB1IP could bind to PTEN (Figure 7A). To test whether LKB1IP directly interact with PTEN under stimulated condition, we performed coimmunoprecipitation assays in NRCMs. The interaction of LKB1IP and PTEN was increased after ISO treatment for 15 min (Figure 7B). Immunohistochemistry against PTEN and LKB1IP in the heart tissues from mouse pathological hypertrophy were performed, which further validated their interaction (Supplementary Figure 7). Importantly, basal and ISO‐induced phosphatase activity of PTEN was significantly reduced with LKB1IP overexpression (Figure 7C). However, LKB1IP overexpression did not affect PTEN protein level (Supplementary Figure 5). Thus, the phosphatase activity of PTEN but not protein level was regulated by LKB1IP. To further confirm that LKB1IP regulates Akt phosphorylation via PTEN, NRCMs were overexpressed with LKB1IP and/or PTEN followed by ISO treatment. LKB1IP overexpression enhanced Akt phosphorylation, which was suppressed by PTEN overexpression (Figure 7D), which suggests that LKB1IP promotes PTEN‐mediated Akt phosphorylation. Taken together, these data demonstrate that LKB1IP may positively activate Akt signalling by directly targeting PTEN and subsequently inhibit its phosphatase activity (Figure 7E).

## 4 DISCUSSION

In this study, LKB1IP expression was up**‐**regulated under pathological cardiac hypertrophy in humans and mice. LKB1IP^‐/‐^ mice were less vulnerable to the pathological cardiac remodelling induced by ISO or TAC. In addition, overexpression of LKB1IP aggravated ISO‐induced cardiomyocyte hypertrophy, and knockdown of LKB1IP ameliorated ISO‐induced hypertrophy. Mechanistically, LKB1IP positively activated Akt signalling by directly targeting PTEN and then inhibiting its phosphatase activity.

LKB1 is a tumour suppressor and a serine/threonine kinase that phosphorylates AMPK family members.^12^ Peutz‐Jeghers syndrome is characterized by the accumulation of non‐cancerous gastrointestinal polyps and increased risk of cancer; LKB1 germline mutations occur in patients with this disease.^13^ In our previous study, we found that endothelial cell‐specific LKB1 knockout led to hypertension and increased angiogenesis in mice.^14^, ^15^ LKB1IP was first identified in 2001 as an LKB1‐interacting protein consisting of 25 exons, mapping to human chromosome 2q36 and encoding a protein of 121 KDa.^8^ LKB1IP could interact with the transforming growth factor β (TGFβ)‐regulated transcription factor SMAD4, forming a LKB1‐LKB1IP‐SMAD4 ternary complex, which results in TGFβ signalling inhibition.^9^ However, much less is known about other functions of LKB1IP. In this study, we generated LKB1IP‐knockout mice to explore its function. LKB1IP deletion did not affect the expression of LKB1 and AMPKα phosphorylation in the mouse heart, which suggests that LKB1IP may play a role independent of LKB1 and AMPK. The highlight of the study is that LKB1IP was first identified as a new regulator of cardiac hypertrophy, which broadens the biological functions of LKB1IP.

PTEN is a phosphatase that can act on both polypeptide and phosphoinositide substrates, which are frequently disrupted in multiple sporadic tumours and targeted by germline mutations in patients with cancer predisposition syndromes.^6^ Specifically, loss of PTEN function would increase cellular levels of PIP3, thereby enhancing the activation of Akt.^7^ PTEN expression is regulated by many transcription factors such as the zinc‐finger transcription factor sal‐like protein 4^16^ and the transacting EMT transcription factor SNAIL (also known as SNAI1)^17^ as well as microRNAs such as miR‑19 in Cowden and leukaemia disease,^18^, ^19^ the miR‑17‐92 cluster in lymphoproliferative disease,^20^ miR‑21 in multiple cancers and metabolic and inflammatory diseases.^21^, ^22^ PTEN function can be modulated by its interacting proteins such as Na^+^/H^+^ exchanger regulatory factor (also known as SLC9A3R1),^23^ membrane‐associated guanylate kinase inverted 2,^24^ β‑arrestins,^25^ motor protein myosin V,^26^ PtdIns(3,4,5)P3‐dependent RAC exchanger factor 2a,^27^ shank‐interacting protein‐like 1 (also known as SHARPIN)^28^ and α‑mannosidase 2C1.^29^ During the development of cardiac hypertrophy, PTEN is modulated and plays a vital role. Suppression of PTEN by miR‐301a could promote cardiomyocytes proliferation.^30^ Tripartite motif 10 (TRIM10) promoted ubiquitination of PTEN, consequently resulting in its proteasomal degradation and activation of hypertrophic signalling.^31^ Besides, the 70 kDa isoform of PTEN, PTEN‐L, interaction with MFN1 on the mitochondria, could cause cardiomyocyte apoptosis during ethanol toxicity in the heart. Inhibition of PTEN activity prevented mitochondrial PTEN‐L‐MFN1 interaction for organelle dysfunction, conferring resistance to alcohol‐induced toxicity.^32^ In this study, we demonstrated that LKB1IP could bind with PTEN and inhibit its phosphatase activity. Thus, LKB1IP may function as a new inhibitor of PTEN. This discovery uncovered a novel regulatory mechanism of PTEN activity in cardiac hypertrophy.

Cardiomyocytes proliferate rapidly during embryogenesis but lose their proliferative capacity soon after birth.^33^, ^34^ However, adult cardiomyocytes retain the ability to respond to a few common disease stimuli including myocardial infarction or ischaemia associated with coronary artery disease, chronic hypertension, valvular insufficiency and stenosis, myocarditis, congenital malformations, familial hypertrophic and dilated cardiomyopathies, and diabetic cardiomyopathy.^35^, ^36^, ^37^ Over time, individuals with cardiac hypertrophy, which contributes to cardiac remodelling including hypertrophic growth and interstitial expansion, are vulnerable to heart failure, arrhythmia or sudden death.^34^ Thus, the detailed mechanism in cardiac hypertrophy needs exploration.

The PI3K/Akt pathway is one of the most important regulatory signalling pathways in cardiac hypertrophy. Once phosphorylated by PIP3, Akt is activated and phosphorylates downstream targets, which results in cardiac hypertrophy.^5^ In this study, LKB1IP expression was up**‐**regulated under pathological cardiac hypertrophy in humans and mice. LKB1IP knockout alleviated ISO‐ or TAC‐induced hypertrophy. Mechanistically, LKB1IP may positively activate Akt signalling by directly targeting PTEN. Thus, LKB1IP plays an important role in the development of pathological cardiac hypertrophy.

In summary, this study provides both in vivo and in vitro evidence that LKB1IP functions as a novel positive regulator of pathological cardiac hypertrophy. These findings improve our understanding of the mechanism of pathological cardiac hypertrophy and suggest that LKB1IP may be a therapeutic target for ameliorating pathological cardiac hypertrophy.

## CONFLICT OF INTEREST

The authors confirm that there are no conflicts of interest.

## AUTHORS CONTRIBUTION

MT and XJ: Design and research. XL and JY: Data. YZ, CZ and WZ: Conceptualization, reviewing and writing. All authors: Discussed and comment.

## Supporting information

Supplementary Material

## References

[1] Benjamin EJ , Virani SS , Callaway CW , et al. Heart disease and stroke statistics‐2018 update: a report from the American Heart Association. Circulation. 2018;137(12):e467‐e492. 10.1161/CIR.0000000000000558 29386200

[2] Braunwald E . The war against heart failure: the Lancet lecture. The Lancet. 2015;385(9970):812‐824. 10.1016/S0140-6736(14)61889-4 25467564

[3] Shimizu I , Minamino T . Physiological and pathological cardiac hypertrophy. J Mol Cell Cardiol. 2016;97:245‐262. 27262674 10.1016/j.yjmcc.2016.06.001

[4] van Berlo JH , Maillet M , Molkentin JD . Signaling effectors underlying pathologic growth and remodeling of the heart. J Clin Invest. 2013;123(1):37‐45. 23281408 10.1172/JCI62839 PMC3533272

[5] Morisco C , Zebrowski D , Condorelli G , Tsichlis P , Vatner SF , Sadoshima J . The Akt‐glycogen synthase kinase 3beta pathway regulates transcription of atrial natriuretic factor induced by beta ‐adrenergic receptor stimulation in cardiac myocytes. J Biol Chem. 2000;275(19):14466‐14475. 10799529 10.1074/jbc.275.19.14466

[6] Song MS , Salmena L , Pandolfi PP . The functions and regulation of the PTEN tumour suppressor. Nat Rev Mol Cell Biol. 2012;13(5):283‐296. 22473468 10.1038/nrm3330

[7] Crackower MA , Oudit GY , Kozieradzki I , et al. Regulation of myocardial contractility and cell size by distinct PI3K‐PTEN signaling. Pathways. 2002;110(6):737‐749. 10.1016/s0092-8674(02)00969-8 12297047

[8] Smith DP , Rayter SI , Niederlander C , et al. LIP1, a cytoplasmic protein functionally linked to the Peutz‐Jeghers syndrome kinase LKB1. Hum Mol Genet. 2001;10(25):2869‐2877. 11741830 10.1093/hmg/10.25.2869

[9] Moren A , Raja E , Heldin CH , Moustakas A . Negative regulation of TGFbeta signaling by the kinase LKB1 and the scaffolding protein LIP1. J Biol Chem. 2011;286(1):341‐353. 20974850 10.1074/jbc.M110.190660 PMC3012991

[10] Li H , He C , Feng J , et al. Regulator of G protein signaling 5 protects against cardiac hypertrophy and fibrosis during biomechanical stress of pressure overload. Proc Natl Acad Sci U S A. 2010;107(31):13818‐13823. 20643937 10.1073/pnas.1008397107 PMC2922261

[11] Hohimer AR , Davis LE , Hatton DC . Repeated daily injections and osmotic pump infusion of isoproterenol cause similar increases in cardiac mass but have different effects on blood pressure. Can J Physiol Pharmacol. 2005;83(2):191‐197. 15791293 10.1139/y04-137

[12] Shaw RJ , Bardeesy N , Manning BD , et al. The LKB1 tumor suppressor negatively regulates mTOR signaling. Cancer Cell. 2004;6(1):91‐99. 15261145 10.1016/j.ccr.2004.06.007

[13] Avizienyte E , Loukola A , Roth S , et al. LKB1 somatic mutations in sporadic tumors. Am J Pathol. 1999;154(3):677‐681. 10079245 10.1016/S0002-9440(10)65314-X PMC1868601

[14] Zhang W , Ding Y , Zhang C , et al. Deletion of endothelial cell‐specific liver kinase B1 increases angiogenesis and tumor growth via vascular endothelial growth factor. Oncogene. 2017;36(30):4277‐4287. 28346429 10.1038/onc.2017.61 PMC5532072

[15] Zhang W , Wang Q , Wu Y , et al. Endothelial cell‐specific liver kinase B1 deletion causes endothelial dysfunction and hypertension in mice in vivo. Circulation. 2014;129(13):1428‐1439. 24637557 10.1161/CIRCULATIONAHA.113.004146 PMC3972325

[16] Lu J , Jeong H , Kong N , et al. Stem cell factor SALL4 represses the transcriptions of PTEN and SALL1 through an epigenetic repressor complex. PLoS One. 2009;4(5):e5577. 19440552 10.1371/journal.pone.0005577 PMC2679146

[17] Escrivà M , Peiró S , Herranz Nicolás , et al. Repression of PTEN phosphatase by Snail1 transcriptional factor during gamma radiation‐induced apoptosis. Mol Cell Biol. 2008;28(5):1528‐1540. 18172008 10.1128/MCB.02061-07 PMC2258777

[18] Mavrakis KJ , Wolfe AL , Oricchio E , et al. Genome‐wide RNA‐mediated interference screen identifies miR‐19 targets in Notch‐induced T‐cell acute lymphoblastic leukaemia. Nat Cell Biol. 2010;12(4):372‐379. 20190740 10.1038/ncb2037 PMC2989719

[19] Olive V , Bennett MJ , Walker JC , et al. miR‐19 is a key oncogenic component of mir‐17‐92. Genes Dev. 2009;23(24):2839‐2849. 20008935 10.1101/gad.1861409 PMC2800084

[20] Xiao C , Srinivasan L , Calado DP , et al. Lymphoproliferative disease and autoimmunity in mice with increased miR‐17‐92 expression in lymphocytes. Nat Immunol. 2008;9(4):405‐414. 18327259 10.1038/ni1575 PMC2533767

[21] Ma X , Kumar M , Choudhury SN , et al. Loss of the miR‐21 allele elevates the expression of its target genes and reduces tumorigenesis. Proc Natl Acad Sci U S A. 2011;108(25):10144‐10149. 21646541 10.1073/pnas.1103735108 PMC3121848

[22] Meng F , Henson R , Wehbe‐Janek H , Ghoshal K , Jacob ST , Patel T . MicroRNA‐21 regulates expression of the PTEN tumor suppressor gene in human hepatocellular cancer. Gastroenterology. 2007;133(2):647‐658. 17681183 10.1053/j.gastro.2007.05.022 PMC4285346

[23] Takahashi Y , Morales FC , Kreimann EL , Georgescu MM . PTEN tumor suppressor associates with NHERF proteins to attenuate PDGF receptor signaling. EMBO J. 2006;25(4):910‐920. 16456542 10.1038/sj.emboj.7600979 PMC1383560

[24] Lima‐Fernandes E , Enslen H , Camand E , et al. Distinct functional outputs of PTEN signalling are controlled by dynamic association with beta‐arrestins. EMBO J. 2011;30(13):2557‐2568. 21642958 10.1038/emboj.2011.178 PMC3155309

[25] Wu X , Hepner K , Castelino‐Prabhu S , et al. Evidence for regulation of the PTEN tumor suppressor by a membrane‐localized multi‐PDZ domain containing scaffold protein MAGI‐2. Proc Natl Acad Sci U S A. 2000;97(8):4233‐4238. 10760291 10.1073/pnas.97.8.4233 PMC18208

[26] van Diepen MT , Parsons M , Downes CP , Leslie NR , Hindges R , Eickholt BJ . MyosinV controls PTEN function and neuronal cell size. Nat Cell Biol. 2009;11(10):1191‐1196. 19767745 10.1038/ncb1961 PMC2756284

[27] Fine B , Hodakoski C , Koujak S , et al. Activation of the PI3K pathway in cancer through inhibition of PTEN by exchange factor P‐REX2a. Science. 2009;325(5945):1261‐1265. 19729658 10.1126/science.1173569 PMC2936784

[28] He L , Ingram A , Rybak AP , Tang D . Shank‐interacting protein‐like 1 promotes tumorigenesis via PTEN inhibition in human tumor cells. J Clin Invest. 2010;120(6):2094‐2108. 20458142 10.1172/JCI40778 PMC2877943

[29] He L , Fan C , Kapoor A , et al. alpha‐Mannosidase 2C1 attenuates PTEN function in prostate cancer cells. Nat Commun. 2011;2:307. 21556061 10.1038/ncomms1309

[30] Zhen L , Zhao Q , Lü J , et al. miR‐301a‐PTEN‐AKT signaling induces cardiomyocyte proliferation and promotes cardiac repair post‐MI. Mol Ther Nucleic Acids. 2020;22:251‐262. 33230431 10.1016/j.omtn.2020.08.033 PMC7515978

[31] Yang H , Wang XX , Zhou CY , et al. Tripartite motif 10 regulates cardiac hypertrophy by targeting the PTEN/AKT pathway. J Cell Mol Med. 2020;24(11):6233‐6241. 32343488 10.1111/jcmm.15257 PMC7294125

[32] Sivakumar A , Shanmugarajan S , Subbiah R , et al. Cardiac Mitochondrial PTEN‐L determines cell fate between apoptosis and survival during chronic alcohol consumption. Apoptosis. 2020;25(7–8):590‐604. 32591959 10.1007/s10495-020-01616-2

[33] Li F , Wang X , Gerdes AM . Formation of binucleated cardiac myocytes in rat heart: II. Cytoskeletal organisation. 1997;29(6):1541‐1551. 10.1006/jmcc.1997.0403 9220341

[34] Berenji K , Drazner MH , Rothermel BA , Hill JA . Does load‐induced ventricular hypertrophy progress to systolic heart failure? Am J Physiol Heart Circ Physiol. 2005;289(1):H8‐H16. 15961379 10.1152/ajpheart.01303.2004

[35] Ahuja P , Sdek P , MacLellan WR . Cardiac myocyte cell cycle control in development, disease, and regeneration. Physiol Rev. 2007;87(2):521‐544. 17429040 10.1152/physrev.00032.2006 PMC2708177

[36] Klein L , O’Connor CM , Gattis WA , et al. Pharmacologic therapy for patients with chronic heart failure and reduced systolic function: review of trials and practical considerations. Am J Cardiol. 2003;91(9A):18‐40. 10.1016/s0002-9149(02)03336-2 12729848

[37] Lips DJ , deWindt LJ , van Kraaij DJ , Doevendans PA . Molecular determinants of myocardial hypertrophy and failure: alternative pathways for beneficial and maladaptive hypertrophy. Eur Heart J. 2003;24(10):883‐896. 12714020 10.1016/s0195-668x(02)00829-1

