#  

**DOI:** 10.1111/jcmm.17194

**Published:** 2022-03-06

In Tian M et al.,^1^ the published article contains errors in Figure 3D and Figure 6D. The correct figures are shown below. The authors confirm all results and conclusions of this article remain unchanged.

**FIGURE 1:**
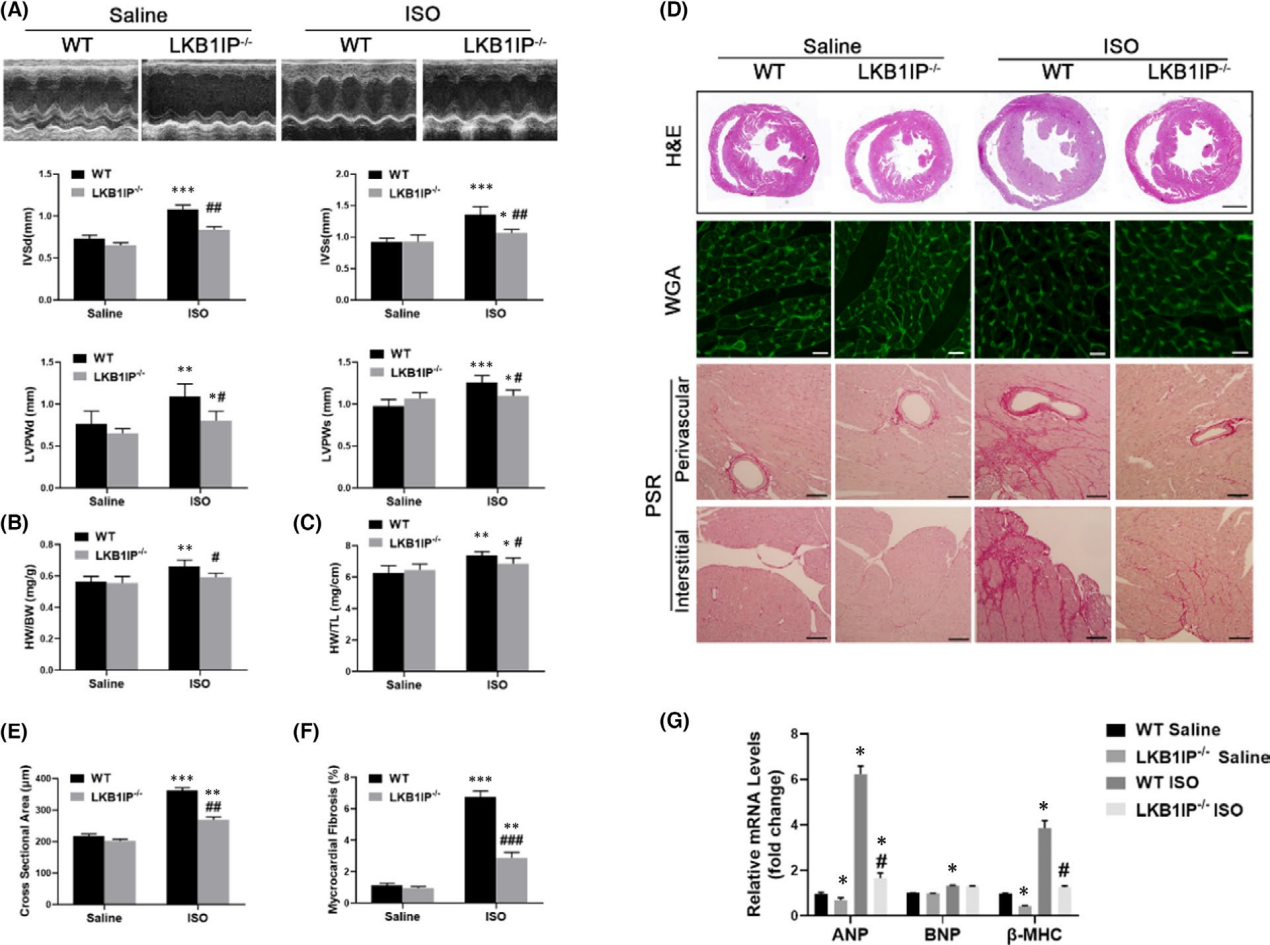
LKB1IP deficiency alleviates ISO‐induced cardiac hypertrophy. (A) Representative M‐mode echocardiography of the left ventricle (top). Measurement of diastolic interventricular septal thickness (IVSd), systolic interventricular septal thickness (IVSs), diastolic left ventricular posterior wall (LVPWd) and systolic left ventricular posterior wall (LVPWs) in the indicated groups (n = 5). *p < 0.05, **p < 0.01 ***p < 0.001 vs. WT Saline; ^#^ *p <* 0.05, ^##^ *p <* 0.01 vs. WT ISO. (B) Quantitative analysis of ratio of heart weight to body weight (HW/BW) in the indicated groups (*n* = 5). **p < 0.01 vs. WT Saline; ^#^ *p* < 0.05 *vs*. WT ISO. (C) Quantitative analysis of ratio of HW to tibial length (TL) in the indicated groups (n=5). *p < 0.05, **p < 0.05 vs. WT Saline; ^#^ *p* < 0.05 vs. WT ISO. (D) Analysis of whole hearts (the first row; scale bar, 1000 μm) and heart sections stained with wheat germ agglutinin (WGA; the second row; scale bar, 50 μm) or picosirius red (PSR; the third and fourth rows; scale bars, 50 μm) from the indicated groups 7 days after saline or ISO treatment. (E) Quantitative analysis of the average cardiomyocyte cross‐sectional area in the indicated groups, *n* > 100 cells per group. **p < 0.01, ***p < 0.001 vs. WT Saline; ^##^ *p* < 0.01 vs. WT ISO. (F) Quantitative analysis of left ventricle (LV) collagen volume in the indicated groups, *n* > 15 fields per group. **p < 0.01, *** *p* < 0.001 vs. WT Saline; ^###^ *p* < 0.001 vs. WT ISO. (G) Quantitative PCR analysis of ANP, BNP and β‐MHC mRNA levels in the indicated groups (*n* = 3). *p < 0.05 vs. WT Saline; ^#^ *p* < 0.05 vs. WT ISO

**FIGURE 2:**
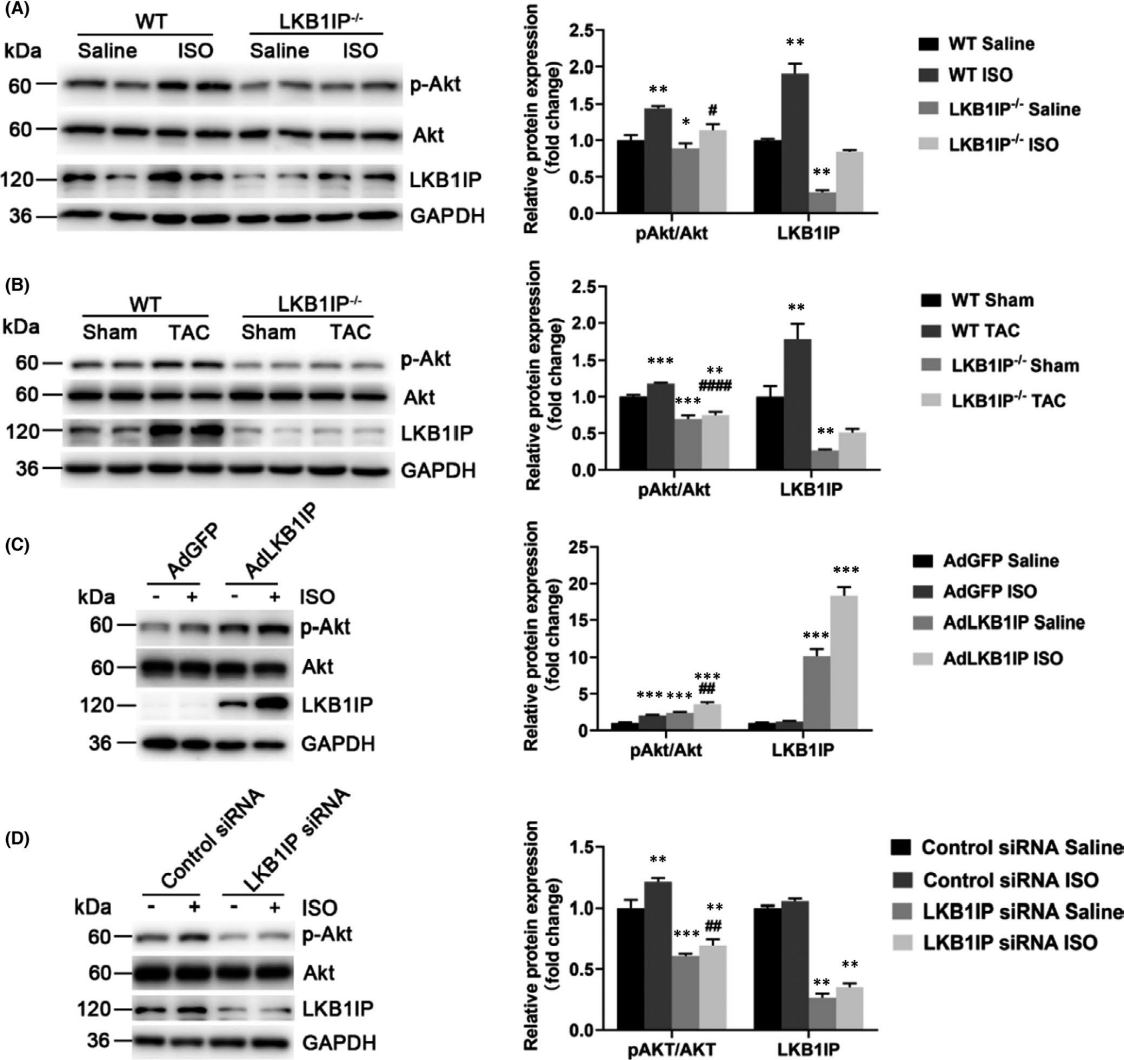
LKB1IP positively regulates Akt phosphorylation. (A) Western blot analysis of phosphorylated Akt from hearts of ISO‐infused WT and LKB1IP^‐/‐^ mice (*n* = 4). *p < 0.05, **p < 0.01 vs. WT Saline; ^#^ *p* < 0.05 vs. WT ISO. (B) Western blot analysis of phosphorylated Akt level in hearts of WT and LKB1IP^‐/‐^ mice after TAC surgery (*n* = 4). **p < 0.01, ***p < 0.001 vs. WT Sham; ^####^ *p* < 0.0001 vs. WT TAC. (C) Western blot analysis of phosphorylated Akt level in NRCMs infected with AdGFP or AdLKB1IP followed by ISO treatment (*n* = 3). ***p < 0.001 vs. AdGFP Saline; ^##^ *p* < 0.01 vs. AdGFP ISO. (D) Western blot analysis of phosphorylated Akt level in NRCMs transfected with control or LKB1IP siRNA followed by ISO treatment (*n* = 3). **p < 0.01, ***p < 0.001 vs. Control siRNA Saline; ^##^ *p* < 0.01 vs. Control siRNA ISO
