# Supplementary material for: LKB1IP promotes pathological cardiac hypertrophy by targeting PTEN/Akt signalling pathway
Source: J Cell Mol Med. 2021 Jan 24;25(5):2517–29. doi: 10.1111/jcmm.16199 (PMC7933949; doi:10.1111/jcmm.16199)
Supplement: Supplementary file 1 — Supplementary Material [file JCMM-25-2517-s001.docx]

**Supplementary material**

**LKB1IP promotes pathological cardiac hypertrophy by targeting PTEN/Akt signaling pathway**

Mi Tian^1^, Xiuxin Jiang^2^, Xinyun Li^1^, Jianmin Yang^1^, Cheng Zhang^1^, Yun Zhang^1^,

Wencheng Zhang^1*^

^1^The Key Laboratory of Cardiovascular Remodeling and Function Research, Chinese Ministry of Education, Chinese National Health Commission and Chinese Academy of Medical Sciences, The State and Shandong Province Joint Key Laboratory of Translational Cardiovascular Medicine, Department of Cardiology, Qilu Hospital of Shandong University, Jinan, China

^2^Department of General Surgery, Qilu Hospital of Shandong University, Jinan, China

**Corresponding author:** Wencheng Zhang, No. 107, Wen Hua Xi Rd, Jinan, Shandong, China. 250012. Phone: 86-531-82169258. Fax: 86-531-82169257.

E-mail: [zhangwencheng@sdu.edu.cn](mailto:zhangwencheng@sdu.edu.cn)

**Supplementary Figure 1.**


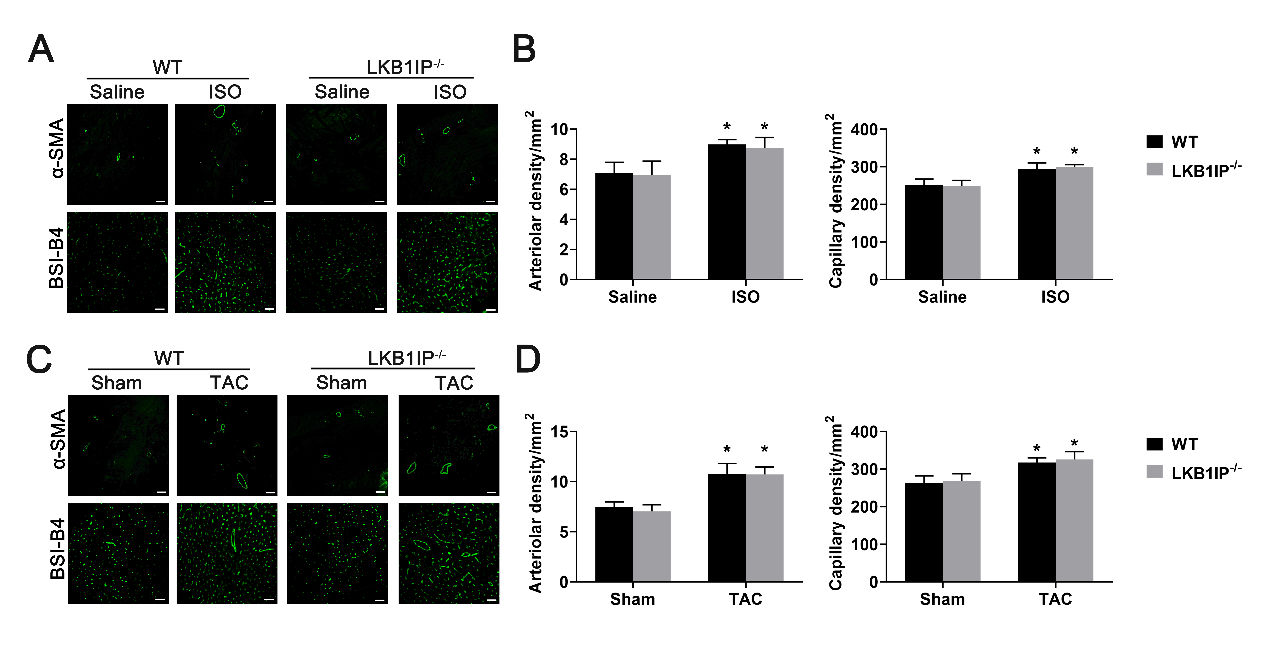


**Supplementary Figure 1. ISO or TAC promotes angiogenesis in heart of mice. (A)(C)** Representative images of BSI-B4 and α-SMA staining in the indicated group. Scale bar, 200 μm for α-SMA, 20 μm for BSI-B4. **(B)(D)** Quantitative analysis of angiogenetic response. Results were indicated by the number of BSI-B4 or α-SMA-positive cells per mm^2^ (n = 3) (**B**)**P* < 0.05 *vs* WT Saline (**D**) **P* < 0.05 *vs* WT Sham.

**Supplementary Figure 2.**


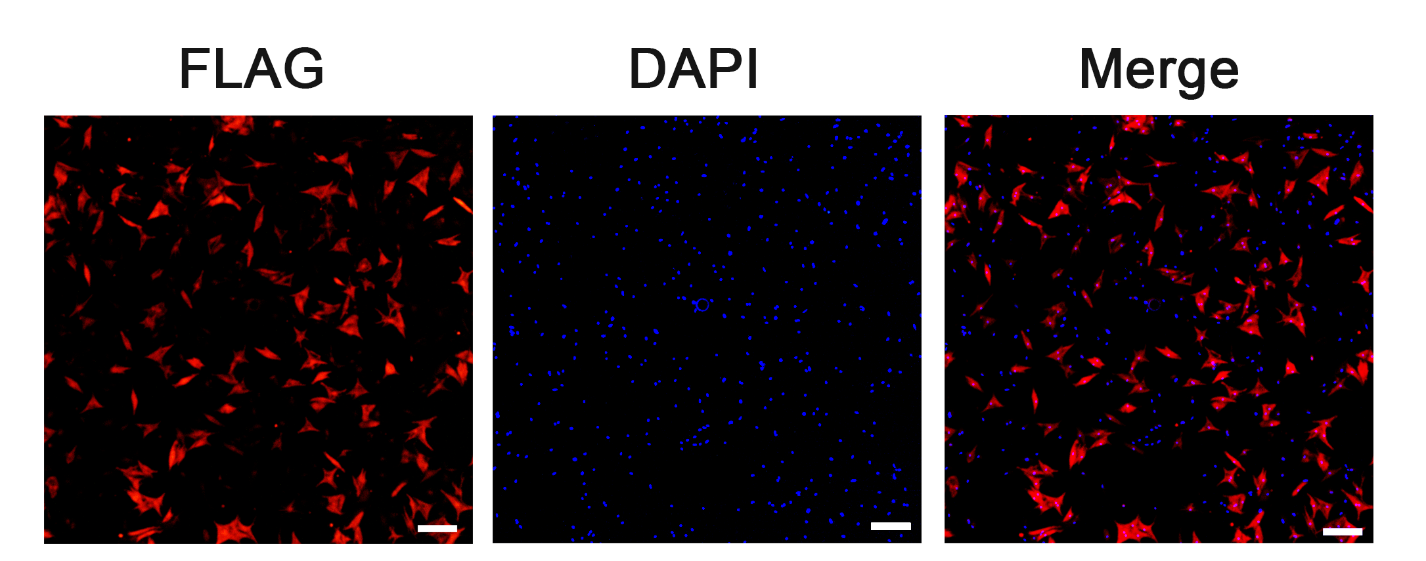


**Supplementary Figure 2.** **Immunohistochemistry against FLAG detecting the efficiency of transduction.** NRCMs were infected with adenovirus expressing FLAG-tagged LKB1IP for 48 hrs followed by immunohistochemistry against FLAG. Scale bar, 200 μm.

**Supplementary Figure 3.**


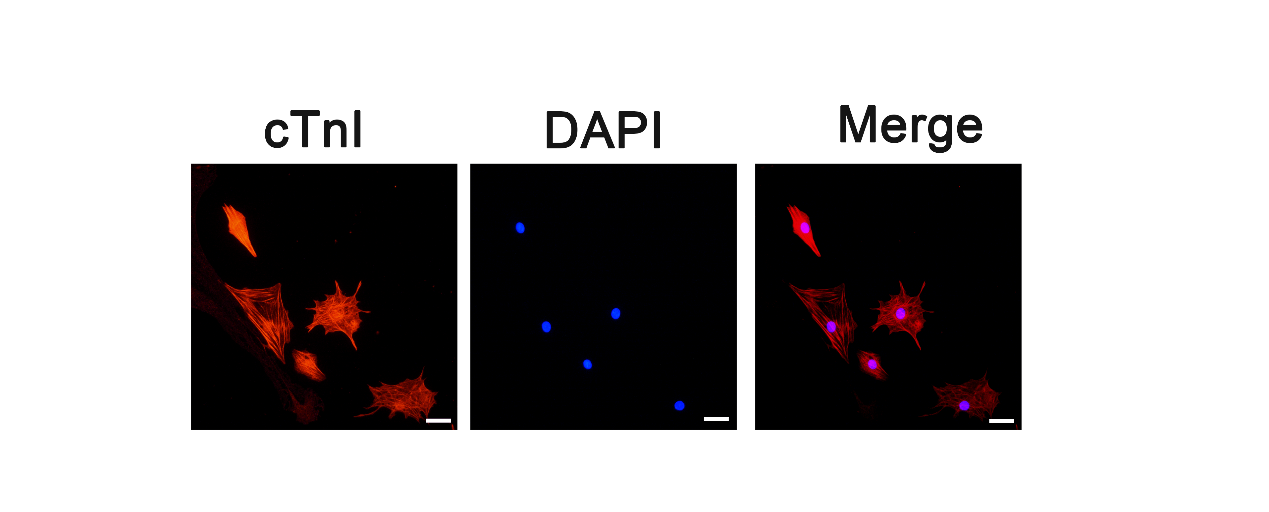


**Supplementary Figure 3. Immunofluorescence against cTnI of primary neonatal rat cardiomyocytes.** Scale bar, 20 μm.

**Supplementary Figure 4.**


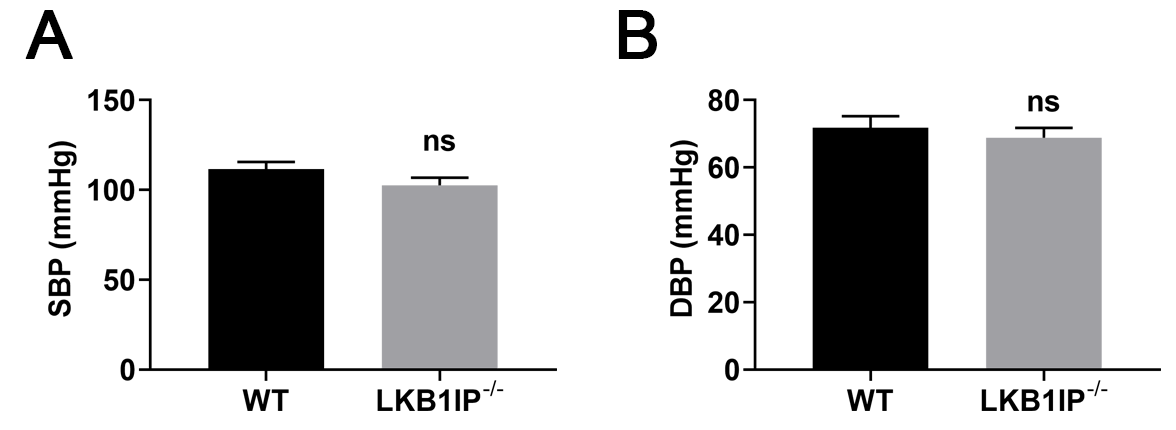


**Supplementary Figure 4.** **Blood pressure in WT and LKB1IP^-/-^ mice. (A)** Systolic blood pressure (SBP) in WT and LKB1IP^-/-^ mice. n=10. **(B)** Diastolic blood pressure (DBP) in WT and LKB1IP^-/-^ mice. n=10.

**Supplementary Figure 5.**


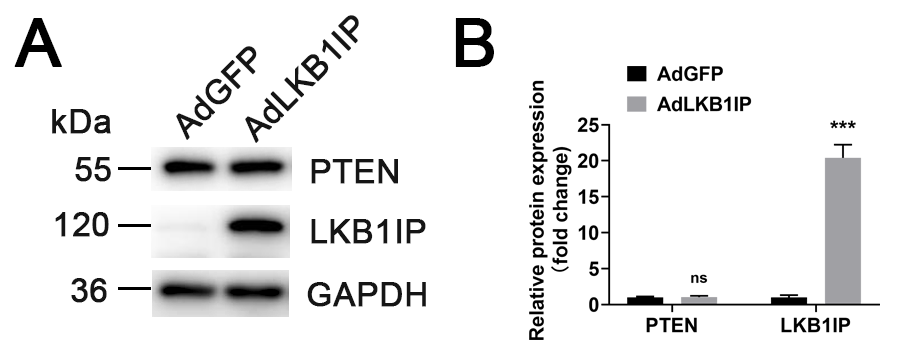


**Supplementary Figure 5. LKB1IP overexpression did not affect PTEN protein level.** **(A)** Western blot analysis of PTEN in NRCMs infected with AdGFP or AdLKB1IP. **(B)** Quantitative analysis of western blot (n=5). ****P*<0.01 *vs* AdGFP.

**Supplementary Figure 6.**


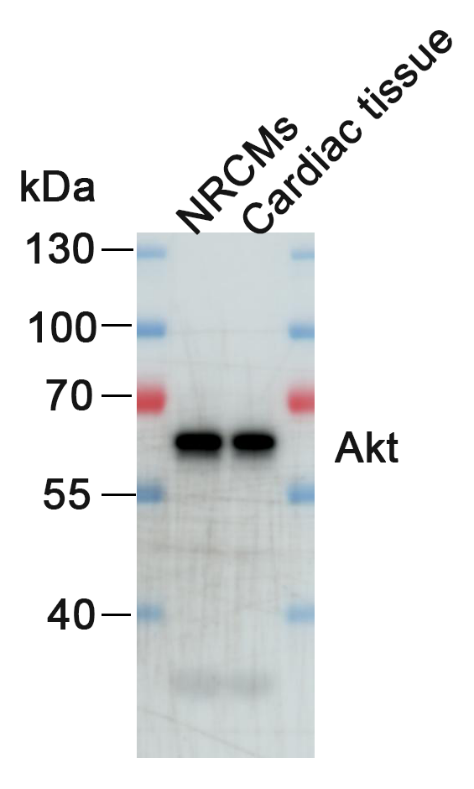


**Supplementary Figure 6. Western blot analysis to detect Akt in NRCMs and mouse cardiac tissue.**

**Supplementary Figure 7.**


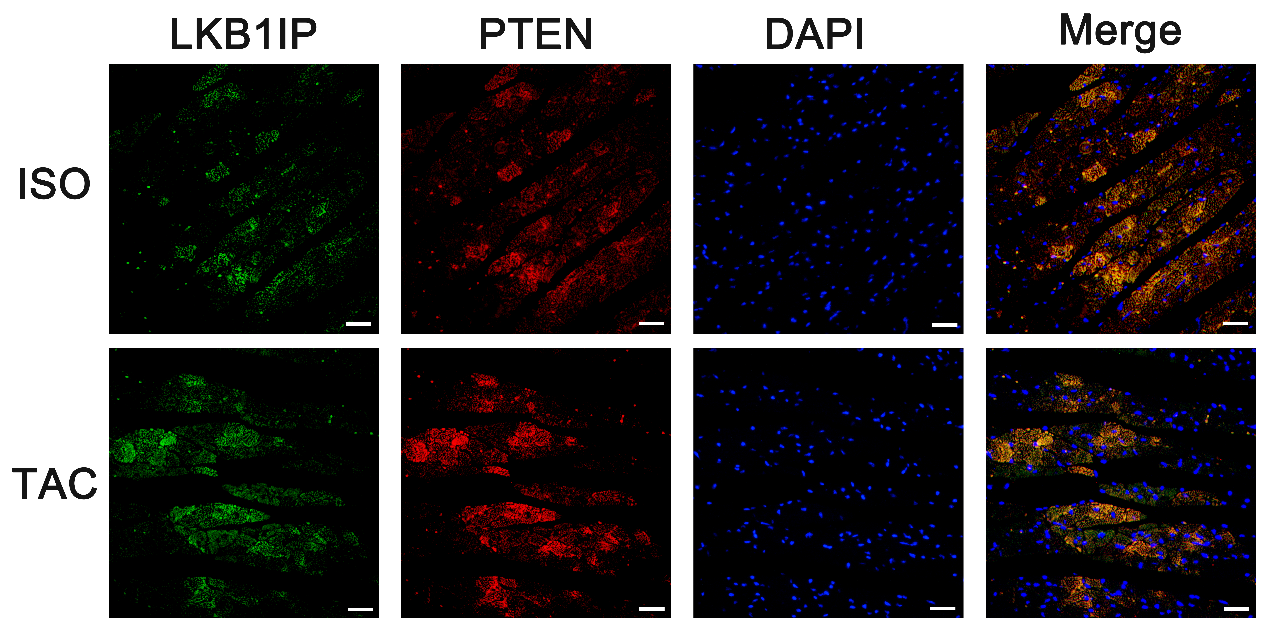


**Supplementary Figure 7. Immunohistochemistry against PTEN and LKB1IP in hearts of mouse pathological hypertrophy.** Scale bar, 20 μm.

**Supplementary Table 1.** **Baseline characteristics of the healthy donors and heart failure patients.**

| Basic information | Heart failure patients  (n=5) | Health donors  (n=5) |
| --- | --- | --- |
| Age(years) | 36±11.9 | 45.4±12.5 |
| Male to female ratio | 3/2 | 2/3 |
| BMI(Kg/m) | 22.44±5.11 | 24.84±3.96 |
| Cardiac function  (NYHA class)  Ⅰ  Ⅱ  Ⅲ  Ⅳ | 0  0  0  5 | __ |
| Duration of the disease(years) | 5±2.5 | __ |
| Type of heart failure  Dilated cardiomyopathy (DCM)  Hypertrophic cardiomyopathy (HCM) | 1  4 | __ |
| Co-morbidities  Metabolic syndrome  COPD (Chronic obstructive pulmonary disease)  Congenital heart disease  Atherosclerosis  hypertension | 2  1  2  1  2 | __  2 |
| Co-medications  ACE-I/ARB  Beta-blockers  Diuretic  Digoxin | 3  4  5  5 | __ |

ACE-I: angiotensin-converting enzyme inhibitors; ARB: angiotensin receptor blocker

**Supplementary Table 2. Echocardiography Parameters in WT and LKB1IP^-/-^ mice at 2 weeks after saline or ISO injection**

|  | Saline | | ISO | |
| --- | --- | --- | --- | --- |
|  | **WT(n=5)** | **LKB1IP^-/-^(n=5)** | **WT(n=5)** | **LKB1IP^-/-^(n=5)** |
| Heart rate | 423±6.75 | 422±6.63 | 426.2±5.71 | 426.6±5.39 |
| LVIDd (mm) | 3.79889±0.253 | 3.8675±0.281 | 3.31319±0.452 | 3.69417±0.322 |
| LVIDs (mm) | 2.98278±0.291 | 2.18833±0.3217 | 2.77694±0.384 | 2.83611±0.247 |
| EF (%) | 50.22456±4.446 | 53.00051±7.937 | 57.4577±7.608 | 54.31668±6.279 |
| FS (%) | 24.99214±4.564 | 28.45999±6.407 | 32.18249±7.265 | 29.53175±9.001 |

LVIDd: diastolic Left ventricular diameter, LVIDs: systolic Left ventricular diameter, EF: Ejection fraction, FS: fraction shortening.

All values are presented as mean ± SEM

**Supplementary Table 3. Echocardiography Parameters in WT and LKB1IP^-/-^ mice at 2 weeks after sham operation or TAC**

|  | Sham | | TAC | |
| --- | --- | --- | --- | --- |
|  | **WT(n=5)** | **LKB1IP^-/-^(n=5)** | **WT(n=5)** | **LKB1IP^-/-^(n=5)** |
| Heart rate | 416±4.71 | 416.6±5.89 | 414.2±6.18 | 410.8±7.22 |
| LVIDd (mm) | 3.72139±0.307 | 3.39083±0.504 | 3.57319±0.144 | 3.50097±0.337 |
| LVIDs (mm) | 2.54944±0.218 | 2.39056±0.377 | 2.68847±0.328 | 2.80042±0.355 |
| EF (%) | 59.47476±7.138 | 57.03087±7.217 | 39.507±2.685^**^ | 39.978±7.642^**^ |
| FS (%) | 31.19001±4.848 | 29.33210±5.209 | 18.941±1.401** | 20.177±4.263** |

** *P*<0.01 *vs.* WT Sham group. All values are presented as mean ± SEM
